# Construction of Rough Surfaces Based on Zirconium Metal–Organic Frameworks to Enhance Photothermal and Photodynamic Therapy for Multiple Myeloma

**DOI:** 10.34133/bmr.0330

**Published:** 2026-02-20

**Authors:** Mengyu Xu, Lihua Ma, Min Liu, Yuanxin Chen, Xianjun Wang, Lijuan Wang, Yanxi Zhu

**Affiliations:** ^1^ Central Laboratory, Linyi People’s Hospital, School of Clinical Medicine, Shandong Second Medical University, Shandong, China.; ^2^ Linyi Key Laboratory of Tumor Biology, Linyi, Shandong, China.; ^3^ Postgraduate Training Base of Linyi People's Hospital, Guangzhou University of Chinese Medicine, Linyi, China.; ^4^ Department of Neurology, Linyi People’s Hospital, Linyi, Shandong, China.

## Abstract

Nanotargeted drug delivery systems (nanotargeted DDS) have emerged as promising solutions to improve treatment precision and reduce toxicity. However, achieving efficient delivery from the bloodstream to the tumor site remains challenging due to the complex tumor microenvironment (TME). To address these issues, a novel pH-responsive surface-switching DDS, UiO@CeO_2_/IR@(bPEI/HA)-A6, was designed. This system features CeO_2_ immobilized on zirconium metal–organic frameworks (UiO-66-NH_2_) to create a rough surface, which is then further modified with bPEI and HA-A6 polymers, facilitating its transport in the blood. After loading the photosensitizer IR-820, its photothermal conversion efficiency reached 26.8%, enabling effective photothermal and photodynamic therapy. The HA-A6 polymer enhanced the targeting effect through receptor-mediated recognition, ensuring more drug accumulation at the tumor site; the rough surface constructed by CeO_2_ increased cell uptake and enhanced endocytosis in cells. The acidic TME exfoliates the coating, exposing the rough surface of CeO_2_, which marked enhances the cellular uptake of DDS, thereby laying a solid foundation for DDS to play an antitumor role. These results indicate that UiO@CeO_2_/IR@(bPEI/HA)-A6 has excellent potential in the treatment of multiple myeloma.

## Introduction

Multiple myeloma is the second most common hematological malignancy after lymphoma [1]. Due to the high relapse rate of myeloma treatment, more frequent administration and high-dose administration are needed, which leads to an increase in drug resistance in patients [2]. Although chemotherapy is the most fundamental treatment for multiple myeloma, there are certain limitations of chemotherapy drugs in the treatment of multiple myeloma, such as poor targeting, systemic toxicity, adverse reactions, and inadequate water solubility of most drugs [3,4]. The precise use of chemotherapy drug concentrations plays a crucial role in evaluating their efficacy and side effects [5]. To improve these situations, a variety of nanotargeted drug delivery systems (nanotargeted DDS) have been synthesized in the past few decades to enhance the efficacy and safety of tumor treatment [6]. It still needs to undergo a lengthy process, from blood circulation to uptake by tumor cells, which requires it to pass through multiple physiological barriers [7]. A large body of literature has demonstrated that the tumor microenvironment (TME) plays a crucial role in the survival, growth, and chemotherapy resistance of multiple myeloma [8]. TME can promote tumor growth and reduce the sensitivity of chemotherapy drugs. To overcome the effect of TME and enhance the targeting and enrichment of drugs at the tumor site, 2 aspects are generally improved. On the one hand, it can reduce its interaction with nontumor tissues in the bloodstream, ensuring that the drug reaches the tumor site efficiently [9]. On the other hand, it can enhance its interaction with tumor tissues, promoting its uptake and retention in tumor cells [10]. A rough surface is a “double-edged sword”, with the advantage that it can enhance the uptake of DDS by tumor cells and increase the specific surface area of the material, thereby enhancing the drug loading capacity and, consequently, better exerting the antitumor effect [11]. Unfortunately, rough surfaces may increase the adhesion of proteins to DDS in the blood circulation, leading to the uptake of DDS by nontumor tissues and thereby weakening the delivery efficiency [12]. To improve the roughness of materials used in blood transport, modification using polymer shells is a standard method for enhancing the surface properties. Branched polyethyleneimine (bPEI) and hyaluronic acid (HA) show good hydrophilicity and biocompatibility [13]. Modification of the polymer shell can not only change the surface roughness of DDS but also encapsulate the drug, preventing leakage during transportation and allowing it to be efficiently transported to the tumor site through the bloodstream [14]. HA and bPEI are pH-responsive and can be stimulated by acidic pH in the TME [15,16], thereby realizing the control of DDS and the precise regulation of drug release.

Metal–organic frameworks (MOFs) exhibit excellent capabilities for drug encapsulation and loading due to their large surface area, high porosity, and large pore size [17,18]. In this study, zirconium metal–organic frameworks (UiO-66-NH_2_) were selected as the carrier for targeted drug delivery due to its excellent water and chemical stability [19,20]. CeO_2_ exhibits a rapid and reversible Ce^3+^/Ce^4+^ cycle, which generates reactive oxygen species (ROS) through various catalytic actions [21]. ROS damages cell membrane integrity and activates apoptotic pathways, leading to cell death [22]. However, the catalytic efficiency of single CeO_2_ in generating ROS is insufficient. According to the collision theory in thermodynamics, increasing the local temperature can enhance the efficiency of Fenton and similar Fenton reactions. By loading the photosensitizer IR-820, the local temperature of the reaction can be increased, which can further promote the generation of ROS by CeO_2_ [23]. Due to the relatively rough surface of UiO@CeO_2_, it has a larger specific surface area and enhanced ability to load IR-820, which can achieve the dual promotion effect of photothermal therapy (PTT) and photodynamic therapy (PDT).

It has been reported that myeloma cells exhibit the characteristic of overexpressed adhesion molecule CD44, which exhibits a high degree of structural heterogeneity and can bind to nucleic acid aptamers with high affinity and strong specificity [24]. Studies have found that A6, a short peptide, can specifically bind to CD44 and enhance the targeting characteristics of materials [25]. It is a good surface modification of DDS. The nanotargeted DDS, constructed by combining the above 2 aspects, can efficiently achieve targeted drug delivery and cellular uptake, resulting in a better antitumor effect [26].

Here, we constructed a tumor acidic microenvironment-responsive rough surface DDS [UiO@CeO_2_/IR@(bPEI/HA)-A6], which consists of UiO-66-NH_2_ as the support, coated with a rough surface CeO_2_ shell and acid-responsive polymer coatings of bPEI and HA-A6. The surface was modified with HA-A6 to increase the targeting of DDS and achieve an efficient delivery effect. UiO@CeO_2_/IR@(bPEI/HA)-A6 has the characteristics of near-infrared (NIR) light-triggered phototherapy through the loading of the new photosensitizer IR-820 and the attributes of CeO_2_ itself, which can produce ROS under the irradiation of 808-nm laser and play a more effective tumor-killing ability. UiO@CeO_2_/IR@(bPEI/HA)-A6 exhibits effective targeting and tumor therapeutic effects in vitro. The rough surface of UiO@CeO_2_/IR@(bPEI/HA)-A6 makes more DDS break through the TME and increase the uptake of tumor cells, which plays a vital role in the treatment of multiple myeloma. Compared with IR-820, UiO@CeO_2_/IR@(bPEI/HA)-A6 cannot be readily metabolized, more accurately targets the tumor site, achieves efficient accumulation in the tumor tissue, and also plays a positive role in tumor killing with good biological safety in vivo.

## Materials and Methods

### Materials

ZrCl_4_, 2-aminoterephthalic acid (NH_2_-BDC), and new indocyanine green (IR-820) were purchased from Shanghai Aladdin Biochemical Technology Co. Ltd. (Shanghai, China). *N*,*N*-dimethylformamide (DMF), hexadecyltrimethylammonium bromide (CTAB), and HA (40 to 100 kDa) were obtained from MACKLIN Biochemical Technology Co. Ltd. (Shanghai, China). Dihydrogen hexachloroplatinate(IV) hexahydrate (H_2_PtCl_6_•6H_2_O) and cerium(III) acetate hydrate [Ce(CH_3_COO)_3_•XH_2_O] were bought from Energy Chemical (Shanghai, China). bPEI was purchased from Sigma-Aldrich (USA). A6 peptide was obtained from China Peptides Co. Ltd. (Shanghai, China). The 1% penicillin and streptomycin RPMI 1640 culture solution, phosphate-buffered saline (PBS), Live/Dead Cell Double Stain Kit, and Reactive Oxygen Species Assay Kit (Red) were purchased from Solarbio (Beijing, China). The Annexin V–fluorescein isothiocyanate (FITC)/propidium iodide (PI) fluorescent double-stain apoptosis detection kit and 10% fetal bovine serum (FBS) were purchased from Pricella (Wuhan, China). The cytometry cycle detection kit was purchased from Bestbio (Shanghai, China). The β-actin antibody is purchased from Huaan Biologics. Bcl-2, Bax, β-actin, and secondary antibodies are purchased from Cell Signaling Technology.

### Preparation of UiO@CeO_2_/IR@(bPEI/HA)-A6

UiO-66-NH_2_ was synthesized as previously reported [27]. ZrCl_4_ (40 mg) and NH_2_-BDC (31 mg) were each dissolved in 5 ml of DMF, sonicated vigorously, mixed, followed by 1.7 ml of glacial acetic acid, and sonicated for 30 min, and the final reaction solution was transferred to a reactor and reacted at 120 °C for 24 h. After natural cooling to room temperature, the solution was collected and centrifuged at 11,000 rpm. It was then washed 3 times each with DMF and ethanol and dried in a vacuum oven overnight.

The CeO_2_ shell was partially modified according to previous reports [28]. UiO-66-NH_2_ (200 μg) was dispersed in 11 ml of deionized (DI) water, followed by the addition of CTAB (0.1 M, 0.15 ml) and H_2_PtCl_6_ (1.0 mM, 36 μl), which was prepared on the spot, and then stirred gently for 20 min. Ce(CH_3_COO)_3_•XH_2_O (10 mM, 0.90 ml) was then added, and the solution was maintained at 100 °C for 1 h. The resulting product UiO-66-NH_2_@CeO_2_ (hereinafter referred to as UiO@CeO_2_) was centrifuged at 8,000 rpm and washed 3 times with DI, followed by drying and storage. UiO@CeO_2_ was redispersed by sonication in DI. A certain amount of IR-820 was added, and the mixture was slowly rotated on a turntable at 37 °C in the dark for 12 h until the mixture was completely reactive. After this, it was centrifuged and washed 3 times with DI, obtaining UiO@CeO_2_/IR.

Subsequently, bPEI and HA-A6 were self-assembled in layers onto the surface of UiO@CeO_2_/IR. bPEI (1.0 ml, 1.0 mg/ml) and 50 μg of UiO@CeO_2_/IR, thoroughly and uniformly mixed, reacted for 10 min and centrifuged, and the supernatant was discarded and washed 3 times with DI. The ligation of HA and A6 was carried out according to the reports in the literature [29]. HA (1 mmol) was dissolved in MES (0.2 M,100 ml) solution and stirred at 25 °C, pH 5.0, until completely dissolved, and then 3 mmol 1-(3-dimethylaminopropyl)-3- and 2 mmol 1-(3-dimethylaminopropyl)-3- were added, followed by a drop of 3 mmol of 1-(2-aminoethyl) maleimide (SM-Mal) at pH 5.5, The mixture was stirred at 25 °C for 24 h. After that, the mixture was dialyzed using a dialysis membrane (molecular weight cutoff, 7,000 Da) in acidic DI (pH 3.5) to obtain the HA-Mal product. According to previous reports, 100 mg of HA-Mal and an appropriate A6 peptide (10 mM, 100 μl) were stirred at room temperature for 24 h, and then a Michael addition reaction occurred to obtain HA-A6 [30]. Finally, UiO@CeO_2_/IR@bPEI was stirred with HA-A6 (2.0 mg/ml, 1.0 ml) at the appropriate concentration for 10 min and then centrifuged, and the supernatant was discarded. The complex was washed 3 times with DI to obtain UiO@CeO_2_/IR@(bPEI/HA)-A6.

### Characterization of UiO@CeO_2_/IR@(bPEI/HA)-A6

Each sample was uniformly dissolved in DI and sonicated, then dropped onto a silicon substrate. After drying, the samples were observed under a scanning electron microscope (SEM) of the Hitachi SU8600 model. The samples that have undergone cleaning treatment were subjected to freeze-drying to obtain dry samples, and then observed using the Tecnai G2 F30 S-TWIN type transmission electron microscope (TEM).

### Measurement of the IR-820 standard curve

The IR-820 solution with concentrations of 10, 20, 30, 40, 50, and 60 μg/ml was used to measure its absorbance value at wavelengths ranging from 450 to 900 nm using an ultraviolet–visible (UV–Vis) spectrophotometer. According to the wavelength at its peak, the absorbance values of 15, 20, 25, 30, 35, and 40 μg/ml were measured, and the standard curve equation was drawn.

### Drug loading

IR-820 and UiO@CeO_2_ were dissolved in DI water to prepare solutions of known concentrations. IR-820:UiO@CeO_2_ was mixed at weight ratios of 1:0.5, 1:1, 1:1.5, 1:2, and 1:3. Then, the mixed solution was made up to a total volume of 1 ml, stirred at room temperature for 24 h, centrifuged to obtain the supernatant, and washed 3 times. The supernatant was collected. The absorbance of the solution at 690 nm was measured using a UV–Vis spectrophotometer and then plotted on the IR-820 standard curve to calculate the loading rate of the material, thereby determining the appropriate amount of IR-820 to use. The following formula was used to calculate drug encapsulation efficiency (DEE) and drug loading efficiency (DLE):

$$\begin{array}{c} \begin{array}{c} \begin{array}{c} \mathrm{DEE}\;\left(\%\right)=\left(\left(\text{amount of}\;\mathrm{IR}-820\;\text{delivered}-\text{amount of}\;\mathrm{IR}-820\right.\right.\\ \left.\left.\;\text{in supernatant}\right)/\text{amount of}\;\mathrm{IR}-820\;\text{delivered}\right)\times 100\% \end{array} \end{array} \end{array}$$ (1)

$$\begin{array}{c} \begin{array}{c} \begin{array}{c} \mathrm{DLE}\;\left(\%\right)=\left(\left(\text{amount of}\;\mathrm{IR}-820\;\text{delivered}-\text{amount of}\;\mathrm{IR}-820\right.\right.\\ \left.\;\text{in supernatant}\right)/\left(\text{amount of}\;\mathrm{UiO}@{\mathrm{CeO}}_{2}+\right.\left(\text{amount of}\;\mathrm{IR}-820\right.\\ \left.\left.\;\text{delivered}-\text{amount of}\;\mathrm{IR}-820\;\text{in supernatant}\right)\right)\times 100\% \end{array} \end{array} \end{array}$$ (2)

After determining the feeding ratio, UiO@CeO_2_/IR and UiO@CeO_2_/IR@(bPEI/HA)-A6 were prepared. Subsequently, the UV absorbance curves of IR-820, UiO-66-NH_2_, UiO@CeO_2_, UiO@CeO_2_/IR, and UiO@CeO_2_/IR@(bPEI/HA)-A6 at 625 to 900 nm were detected. A Nicolet iS5 (Thermo Electron Scientific Instruments Corp.) was used to analyze the Fourier transform infrared spectrum of the relationship between the loading of UiO@CeO_2_ and IR-820.

### Particle size and zeta potential

The size of the material [dynamic light scattering (DLS)] and material charge were detected using a Malvern Zetasizer Ultra Nano Particle Size and Zeta Potential analyzer. Particle size was obtained by SEM analysis.

### Photothermal performance testing

To evaluate the photothermal conversion efficiency of the material, UiO@CeO_2_/IR@(bPEI/HA)-A6 (100 μg/ml, 1 ml) was placed in a 12-well plate and irradiated (1 W/cm^2^) using a cell phototoxicity meter, and the heating and cooling curves of the material were measured using a K-type thermocouple thermometer.

To determine the dependence of material concentration on photothermal efficiency, 1 ml of UiO@CeO_2_/IR@(bPEI/HA)-A6 in water dispersion at different concentrations (40, 60, 80, 100, and 120 μg/ml) was irradiated with 808 nm of 1 W/cm^2^ laser. A K-type thermocouple thermometer was used to measure the temperature curve of the material. To determine the dependence of the photothermal efficiency of the materials irradiated with different power densities, the temperature changes of the same concentration of UiO@CeO_2_/IR@(bPEI/HA)- A6 in DI aqueous solution were analyzed using an 808-nm laser at 0.5, 1.0, 1.5, and 2 W/cm^2^ power densities.

### Cell culture and mouse tumor model

ARH-77 and RPMI8226 myeloma cells were purchased from the Cell Bank of the Chinese Academy of Sciences. The culture conditions consisted of RPMI 1640 medium supplemented with 10% FBS and 1% penicillin–streptomycin. The cells were maintained at 37 °C in an incubator with 5% CO_2_. Six-week-old female BALB/c nude mice were purchased from Jinan Pengyue Experimental Animal Breeding Co. Ltd. (Shandong, China). All nude mice were housed in a standard, specific pathogen-free (SPF) environment. All experiments were approved by the Experimental Animal Management Committee of Linyi People’s Hospital.

### Safety testing of nanotargeted DDS

To evaluate the safety of the nanotargeted DDS in vitro, cell viability was assessed by Cell Counting Kit-8 (CCK8) assay. Different concentrations of UiO@CeO_2_/IR and UiO@CeO_2_/IR@(bPEI/HA)-A6 were prepared to intervene with the cells. ARH-77 cells were seeded into a 96-well cell culture plate, with 100 μl of RPMI 1640 medium and the materials added simultaneously. After 24 h of incubation (5% CO_2_, 37 °C), then CCK-8 solution (10 μl per well) was added to each well. After 1 to 3 h of incubation, the absorbance at 450 nm was measured using a microplate reader. The same procedure was performed for RPMI8226 cells. The cell survival rates under different treatment conditions were calculated accordingly. Cell viability was calculated as follows:

$$\begin{array}{c} \begin{array}{c} \begin{array}{c} \mathrm{cell}\;\text{viability}\;\left(\%\right)=\left({\mathrm{OD}}_{\text{Sample}}-{\mathrm{OD}}_{\text{Blank}}\right)/\\ \left({\mathrm{OD}}_{\text{Negativecontrol}}-{\mathrm{OD}}_{\text{Blank}}\right)\times 100\% \end{array} \end{array} \end{array}$$ (3)

### Tumor-killing ability of nanotargeted DDS

To evaluate the cytotoxicity of the DDS in vitro, cell viability was assessed by the CCK8 assay. Different concentrations of UiO@CeO_2_, UiO@CeO_2_/IR, and UiO@CeO_2_/IR@(bPEI/HA)-A6 mixtures were used to intervene in cells. Then, the treated ARH-77 cells were seeded at a density of 7 × 10^3^ cells per well into a 96-well cell culture plate and 100 μl of fresh RPMI 1640 medium (containing 10% FBS and 1% antibiotics) for 24 h (5% CO_2_, 37 °C). After incubation for 24 h, each well was illuminated with an 808-nm laser (1 W/cm^2^) for 5 min. After illumination, 10 μl of CCK8 solution per well was added to each well, and after 1 to 3 h of incubation, the absorbance at 450 nm was measured using a microplate reader. The same procedure was performed for RPMI8226 cells.

### Live/dead cell staining was used to observe cell viability

The nanotargeted DDS was divided into 2 large groups based on phototoxicity and dark toxicity. In the dark toxicity group, the PBS group was used as the control, and the other 3 groups were treated with mixtures of UiO@CeO_2_, UiO@CeO_2_/IR, and UiO@CeO_2_/IR@(bPEI/HA)-A6 at a concentration of 60 μg/ml. Cells were seeded into 6-well plates at a concentration of 3 × 10^5^ cells/ml and subsequently cultured for 24 h (5% CO_2_, 37 °C). Finally, the cells were harvested by centrifugation and then stained with calcein-AM and PI. Live and dead cells were observed under a fluorescence inverted microscope to compare the safety of different materials. For the detection of phototoxicity of the nanotargeted DDS, the PBS group was used as a control, and the other 3 groups were treated with mixtures of UiO@CeO_2_, UiO@CeO_2_/IR, and UiO@CeO_2_/IR@(bPEI/HA)-A6 at a concentration of 60 μg/ml, respectively. The cells, at a concentration of 3 × 10^5^ cells/ml, were vaccinated in 6-well plates and then incubated (5% CO_2_, 37 °C) for 24 h. After incubation, the cells from the above 4 groups were exposed to an 808-nm laser (1 W/cm^2^) light for 5 min. Upon completion of the illumination, the cells were harvested by centrifugation and then stained with calcein-AM and PI. To detect and compare the killing effect of UiO@CeO_2_/IR@(bPEI/HA)-A6 on ARH-77 cells, live cells and dead cells were observed under a fluorescence inverted microscope.

### Cellular uptake

To further investigate the cellular uptake of the rough surface of the materials, the uptake ability of ARH-77 to the different materials was quantified by flow cytometry. The PBS group was used as the control group, and the other 3 groups were loaded with the same amount of coumarin-6 (C6). The 3 experimental groups were UiO-66-NH_2_/C6, UiO@CeO_2_/C6, and UiO@CeO_2_/C6@(bPEI/HA)-A6. After the above 4 groups were intervened, the cells were seeded in 6-well plates and incubated in serum-free RPMI 1640 medium with 5% CO_2_ at 37 °C for 4 h. Then, the cells were collected, washed with PBS, centrifuged at 1,200 rpm for 5 min, resuspended in 400 μl of PBS, and analyzed by flow cytometry. The entire process was conducted in dark conditions. FlowJo software was used for data analysis.

### Transwell cell uptake experiment

To construct the extracellular microenvironment of tumor cells, a precooled pipette was used to draw the Matrigel, which was then mixed uniformly with serum-free RPMI 1640 medium at a 1:7 ratio. The mixed solution (100 μl) was added vertically into the transwell chamber to spread evenly at the bottom. The chamber was then incubated at 37 °C for 3 h. After 3 h, the unbound Matrigel was carefully aspirated, and the transwell chamber was used to simulate the extracellular microenvironment of tumor cells. Starved cells (5 × 10^3^) were resuspended in 700 μl of complete RPMI 1640 medium containing 10% FBS and inoculated into the wells of a 24-well plate. The transwell chamber was placed on top to construct the overall cell environment. The mixture (100 μl) of 60 μg/ml UiO-66-NH_2_/C6, UiO@CeO_2_/C6, and UiO@CeO_2_/C6@(bPEI/HA)-A6 with the RPMI 1640 medium was added to the upper layer of the transwell chamber. The 24-well plate was placed in a constant-temperature incubator (5% CO_2_, 37 °C) for 24 h. After 24 h, the cells were washed with PBS, and the cell uptake of different materials was observed under a fluorescence inverted microscope.

### In vitro cell apoptosis analysis

Annexin V-FITC/PI double staining was used to analyze the apoptosis of ARH-77 cells. This method was similar to the live/dead cell assay described above. The nanotargeted DDS was divided into 2 large groups based on phototoxicity and dark toxicity. In the dark toxicity group, the PBS group was used as the control, and the other 3 groups were treated with mixtures of UiO@CeO_2_, UiO@CeO_2_/IR, and UiO@CeO_2_/IR@(bPEI/HA)-A6 at a concentration of 60 μg/ml, respectively. Cells were seeded into 6-well plates at a concentration of 3 × 10^5^ cells/ml and subsequently cultured for 24 h (5% CO_2_, 37 °C). For the detection of phototoxicity of the nanotargeted DDS, the PBS group was used as a control, and the other 3 groups were treated with mixtures of UiO@CeO_2_, UiO@CeO_2_/IR, and UiO@CeO_2_/IR@(bPEI/HA)-A6 at a concentration of 60 μg/ml, respectively. The cells were seeded into 6-well plates at a concentration of 3 × 10^5^ cells/ml and subsequently cultured for 24 h (5% CO_2_, 37 °C). After 24 h of incubation, the above 4 groups were illuminated with an 808-nm laser (1 W/cm^2^) for 5 min. Upon completion of the treatment, the cells were harvested by centrifugation and manipulated with FITC and PI reagents before being examined using flow cytometry. FlowJo software was used for analysis.

### Expression level analysis of apoptosis-related proteins

Western blot was used to detect the expression levels of apoptosis-related proteins in ARH-77 cells induced by the nanotargeted DDS. The same grouping and cell treatment methods were used for dark toxicity and phototoxicity as described above. Then, the cells were collected by centrifugation, and the protein was extracted using the lysis buffer. The protein concentration was determined using the BCA Protein Assay Kit, and the loading volume was calculated accordingly. The extracted protein solution was mixed with loading buffer, boiled at 100 °C, and then separated by gel electrophoresis according to molecular weight. The gel was cut, and the membrane was transferred. The film was turned at 250 mA for 45 min, followed by sealing. After completion of blocking, the corresponding primary and secondary antibodies were incubated. The luminescent solution was finally applied to the membrane and exposed using an imager. Grayscale values were collected and analyzed using ImageJ.

### In vitro cell cycle analysis

The cell cycle of ARH-77 cells after different treatments was analyzed using the cell cycle detection kit. Cells were starved in serum-free and antibody-free RPMI 1640 medium for 24 h before being seeded into 6-well plates. The dark toxicity and phototoxicity groups, as well as cell treatment methods, were still carried out. After that, the cells were harvested, fixed with ethanol, and then treated with ribonuclease A and PI stain. Flow cytometry was used to detect the cell cycle after treatment. ModFit software was used to analyze the cell cycle.

### ROS detection

The content of superoxide anion ROS was detected by the dihydroethidium (DHE) red fluorescent dye. The PBS group served as the control group, while the other 3 groups, UiO@CeO_2_, UiO@CeO_2_/IR, and UiO@CeO_2_/IR@(bPEI/HA)-A6, were used as experimental groups to intervene in the cells. The cells were seeded into 6-well plates and subsequently cultured for 24 h (5% CO_2_, 37 °C). After 24 h of incubation, the above 4 groups were illuminated with an 808-nm laser (1 W/cm^2^) for 5 min. Upon completion of the treatment, the cells were harvested by centrifugation and stained using a DHE fluorescent probe, followed by determination of ROS content using flow cytometry. FlowJo software was used for data analysis.

### In vivo biodistribution and tissue fluorescence imaging of nanotargeted DDS

The in vivo imaging system was used to observe the in vivo distribution of the nanotargeted DDS in the tumor site and other organs for evaluation. To establish the multiple myeloma tumor model, mice were irradiated with a linear accelerator (x-rays) at a dose of 3 Gy, and then 200 μl of ARH-77 cell suspension (2 × 10^5^ cells/μl) and Matrigel mixture was injected subcutaneously into the right armpit site of each nude mouse. Due to the fluorescent properties of IR-820, PBS/IR-820 (control group) and UiO@CeO_2_/IR@(bPEI/HA)-A6 (containing IR-820 at a dose of 3 mg/kg) were intravenously injected into mice. The mice were anesthetized at different time points after injection (2, 4, 6, 8, and 10 h), and the fluorescence signals in the mice were detected and photographed by the small animal in vivo imaging system. PBS/IR-820 (control group), UiO@CeO_2_/IR, and UiO@CeO_2_/IR@(bPEI/HA)-A6 (containing IR-820 at a dose of 3 mg/kg) were injected into the mice via the intravenous route. Twenty-four hours after intravenous injection, the mice were anesthetized for in vivo imaging. Subsequently, the tumor-bearing mice were sacrificed, and the main organs (heart, liver, spleen, lung, and kidney) and tumors of the mice were dissected and collected.

### Antitumor activity and biosafety in vivo

The tumor-killing ability of the nanotargeted DDS was tested in an in vivo experiment. When the tumor volume reached a specific size (approximately 200 mm^3^), 20 mice were randomly divided into 4 groups, each with 5 mice, namely, the PBS group, the IR-820 group, the UiO@CeO_2_/IR group, and the UiO@CeO_2_/IR@(bPEI/HA)-A6 group. According to different groups, different drugs (containing IR-820 at a dose of 3 mg/kg) were administered intravenously into the corresponding mice. Eight hours after injection, the tumor sites of the mice were irradiated with an 808-nm laser at 1 W/cm^2^ for 10 min, and the mice received this treatment every 2 d. The above 4 groups were treated with light. The PBS-treated mice were used as the control group. Body weight and tumor volume were recorded every 2 d during treatment. The tumor volume of nude mice was measured using a digital caliper, according to the formula RTV = (*a* × *b*^2^)/2 (mm^3^), where *a* represents the length (mm) and *b* represents the width (mm). After the last treatment, the mice were sacrificed by neck removal and then dissected. The tumors were stripped and photographed. The collected major organs (heart, liver, spleen, lung, and kidney) and tumor tissues were washed with normal saline and fixed in 4% tissue fixative solution for further histological analysis. The above organs and tumor tissues were embedded in paraffin, and paraffin sections were prepared. The paraffin sections of relevant tissues and tumors were stained, sealed, and imaged with a digital microscope (Olympus microscope) to observe their pathological characteristics.

### Statistical analyses

The data were presented as mean ± SD, with *n* = 3 independent treatments, and analyzed statistically using one-way analysis of variance (ANOVA) with Tukey's multiple comparisons test. A *t* test was employed to compare the means of 2 sample groups; *P* < 0.05 was considered statistically significant.

## Results

### Material preparation and characterization

**Fig. 1.**
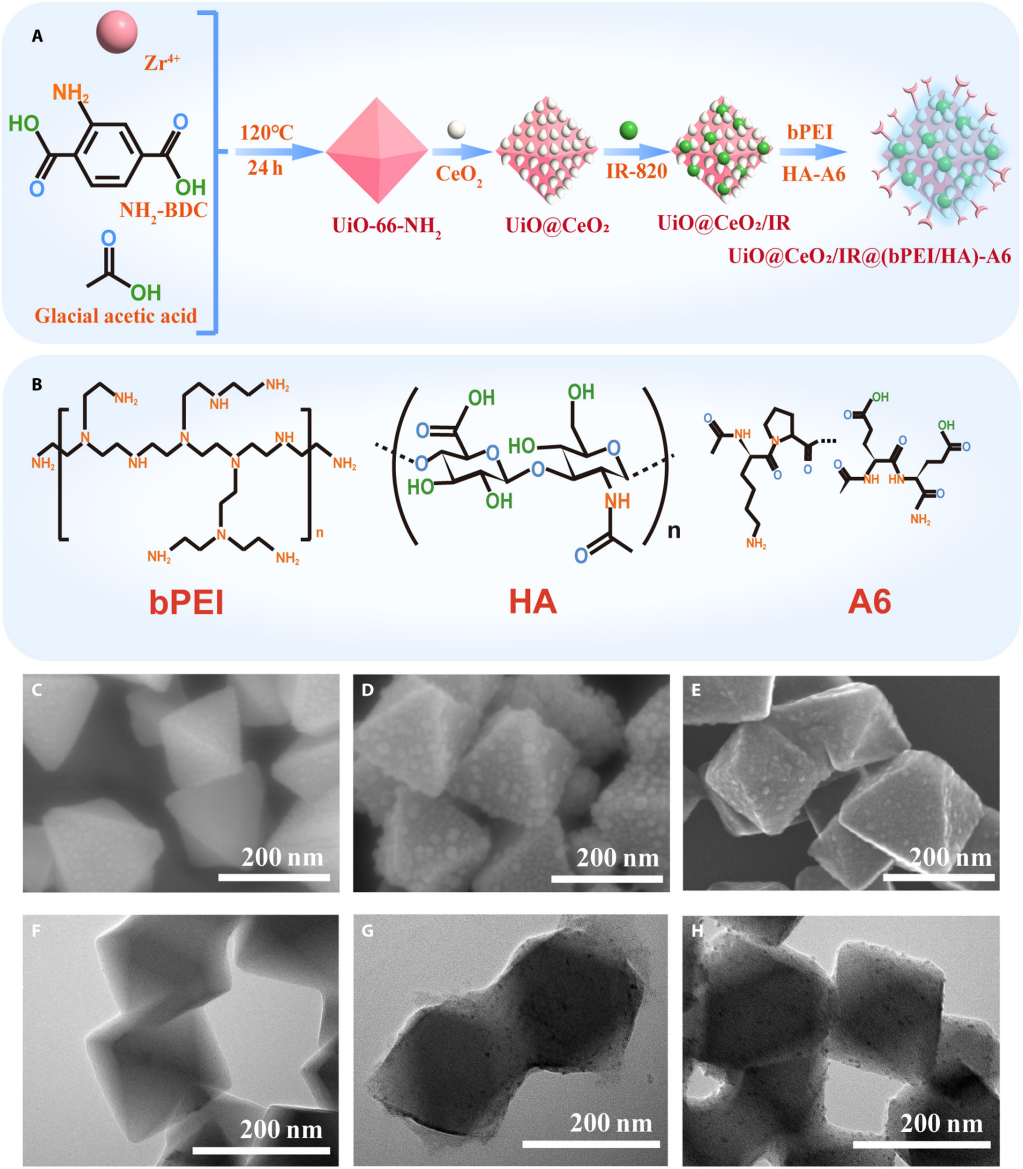
(A) Schematic of the synthesis route for UiO@CeO_2_/IR@(bPEI/HA)-A6. (B) Molecular formula of bPEI, HA, and A6. (C to E) Scanning electron microscope (SEM) images. (C) Image of UiO-66-NH_2_ SEM. (D) Image of UiO@CeO_2_ SEM. (E) Image of UiO@CeO_2_/IR@(bPEI/HA)-A6 SEM. (F to H) Transmission electron microscope (TEM) images. (F) Image of UiO-66-NH_2_ TEM. (G) Image of UiO@CeO_2_ TEM. (H) Image of UiO@CeO_2_/IR@(bPEI/HA)-A6 TEM.

The fabrication of UiO@CeO_2_/IR@(bPEI/HA)-A6 is shown in Fig. 1A. SEM and TEM images show that dispersed UiO-66-NH_2_, UiO@CeO_2_, and UiO@CeO_2_/IR@(bPEI/HA)-A6 nanomaterials have been successfully prepared (Fig. 1C to H). The dispersed UiO-66-NH_2_ showed an octahedral structure with mesopores on its surface. UiO-66-NH_2_ has great potential in drug delivery due to its excellent water and chemical stability.

The surface of UiO@CeO_2_ was obviously uniformly coated with CeO_2_. The rough surface of nanomaterials can enhance adhesion to tumor cells and improve the efficiency of drug delivery [31]. Not only does the attachment of CeO_2_ to UiO-66-NH_2_ improve the delivery efficiency of the material, but CeO_2_ has reversible Ce(III)/Ce(IV) REDOX pairs, and its surface has a large number of oxygen vacancies, allowing them to interact and regulate with free radicals [32,33]. In the acidic pH environment characteristic of cancer cells, CeO_2_ may be cytotoxic and induce cell damage through ROS, further leading to cell death.

The UiO@CeO_2_/IR@(bPEI/HA)-A6 surface is clearly covered by polyelectrolytes and is attached with the targeted substances. The coating of polyelectrolyte blurred the surface of UiO@CeO_2_, and the size of the nanoparticles gradually increased. The use of bPEI/HA modified materials not only prevents the material from being killed by immune cells and drug leakage during blood transportation but also allows bPEI/HA to be lysed under acidic conditions, exposing the rough surface, allowing the material to adhere to the surface of tumor cells. Moreover, the A6 short peptide greatly facilitates bioconjugation and characterization. Modification of UiO@CeO_2_/IR@bPEI/HA with the A6 short peptide can enhance the targeting of the material, achieve more effective drug delivery, and reduce side effects on normal cells.

### Determination of drug loading and IR-820-related properties

**Fig. 2.**
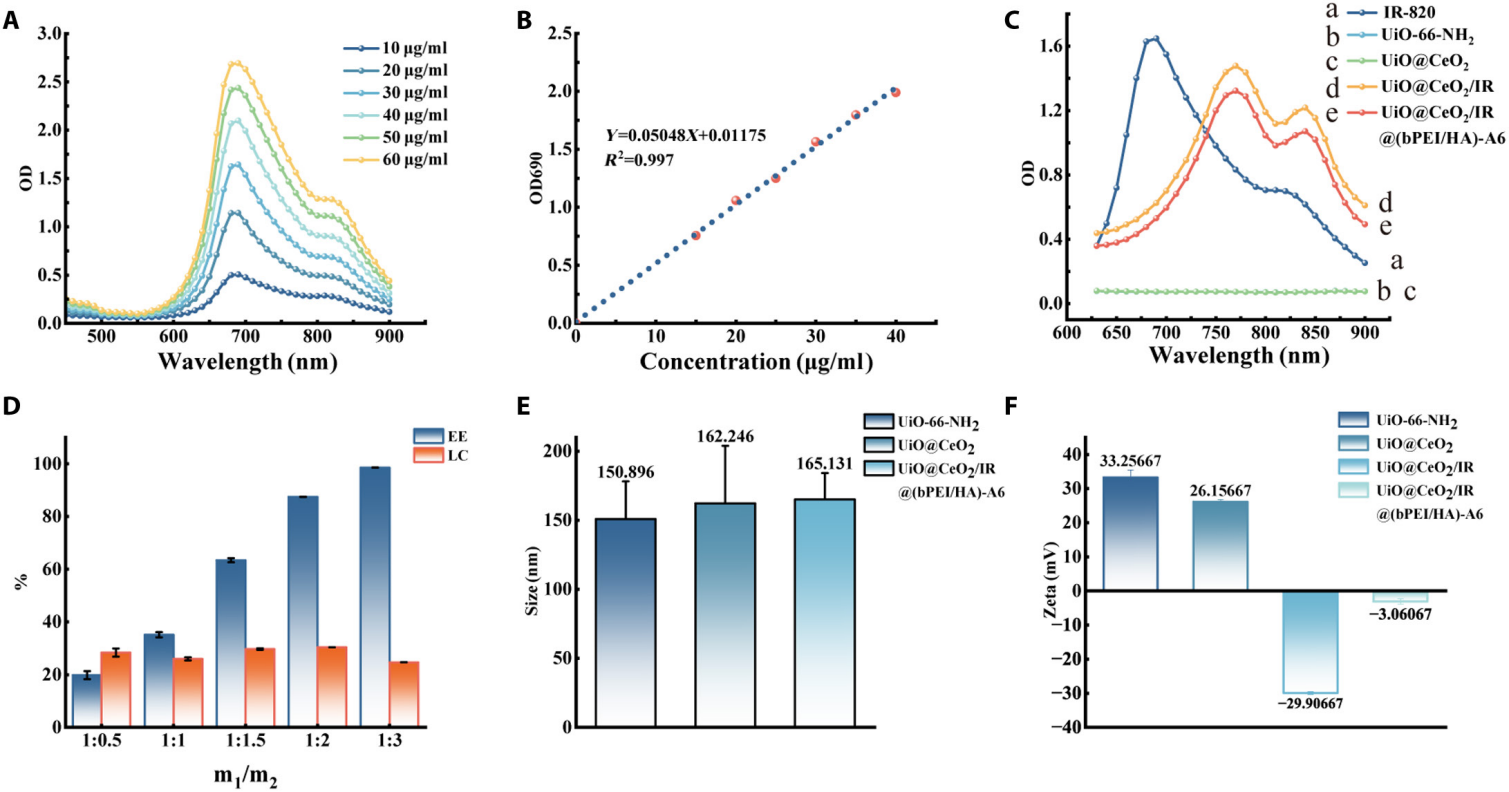
(A) Absorbance analysis of IR-820 at different concentrations. (B) Relationship between the absorbance of different concentrations of IR-820 at 690 nm. The results are as follows: *Y* = 0.05048*X* + 0.01175, *R*^2^ = 0.997, where *Y* is OD690 and *X* is the concentration of IR-820. (C) UV–Vis spectra of IR-820, UiO-66-NH_2_, UiO@CeO_2_, UiO@CeO_2_/IR, and UiO@CeO_2_/IR@(bPEI/HA)-A6. (D) UiO@CeO_2_/IR@(bPEI/HA)-A6 loading capacity (LC) and encapsulation efficiency (EE) for different feeding ratios of IR-820 (m_1_) and UiO@CeO_2_ (m_2_). (E) Particle size of UiO-66-NH_2_, UiO@CeO_2_, and UiO@CeO_2_/IR@(bPEI/HA)-A6. (F) Zeta potential of UiO-66-NH_2_, UiO@CeO_2_, UiO@CeO_2_/IR, and UiO@CeO_2_/IR@(bPEI/HA)-A6.

UV–Vis spectroscopy was used to measure the absorbance values of different concentrations of IR-820 excited at wavelengths ranging from 450 to 900 nm (Fig. 2A). The results showed that the absorbance value of IR-820 was the largest at approximately 690 nm and increased with increasing concentration. According to the absorbance value, the standard curve equation of the drug can be calculated: *Y* = 0.05048*X* + 0.01175, *R*^2^ = 0.997, where *Y* is OD690 and *X* is the concentration of IR-820 (Fig. 2B). According to the standard curve equation, the loading of the drug can be provided as a theoretical basis for the calculation.

The measured UiO-66-NH_2_, UiO@CeO_2_, UiO@CeO_2_/IR, and UiO@CeO_2_/IR@(bPEI/HA)-A6 absorbance peaks are shown: UiO@CeO_2_/IR and UiO@CeO_2_/IR@(bPEI/HA)-A6 have maximum absorption peaks around 770 nm, and UiO-66-NH_2_ and UiO@CeO_2_ have no absorption peaks, so it can be concluded that IR-820 drug loading is successful. The structure of the nanocarrier does not affect the display of the absorption peak. The highest absorption peak is specific. Compared with IR-820 at 690 nm, the red shift phenomenon of UiO@CeO_2_/IR and UiO@CeO_2_/IR@(bPEI/HA)-A6 may be related to the intermolecular interaction in the UiO@CeO_2_ channel or the influence of solvent [34] (Fig. 2C).

The encapsulation efficiency and loading efficiency of IR-820 are significant indications for PTT and PDT. In this experiment, the loading rate and encapsulation rate of the material were calculated according to the different feeding ratios of IR-820 (m_1_) and UiO@CeO_2_ (m_2_). The results show that the encapsulation rate and loading rate vary with different feeding ratios. With the increase in the amount of IR-820, the encapsulation rate increased gradually, while the load rate initially increased and then decreased. When the m_1_/m_2_ ratio is 1/2, the effect of encapsulation rate and load rate is the best. The average encapsulation efficiency and loading efficiency of UiO@CeO_2_/IR@(bPEI/HA)-A6 were 87.47 ± 0.06% and 30.43 ± 0.01%, respectively (Fig. 2D). This demonstrated the excellent drug loading performance of UiO-66-NH_2_ as MOFs. In addition, the rough surface area also contributed to the high DLE. Additionally, the surface coating (bPEI/HA)-A6 reduces the likelihood of drug leakage.

To further evaluate the interaction between UiO@CeO_2_ and IR-820, we measured the Fourier transform infrared spectra of UiO@CeO_2_ and UiO@CeO_2_/IR (Fig. S1). In the infrared spectrum, the absorption at 1,658 cm^−1^ in UiO@CeO_2_/IR corresponds to the stretching vibration of the intramolecular double bond (C=C) of the molecule. However, there is no such peak in the UiO@CeO_2_ complex, indicating that IR-820 is bound to UiO@CeO_2_ through double bond-related interactions. The bending vibration peak of the framework at 483 cm^−1^ exists in both, indicating that the loading process did not damage the main framework structure of UiO@CeO_2_ but achieved the loading of IR-820 through the interaction between functional groups.

### Particle size and zeta potential

The size of the nanotargeted DDS was determined by SEM, that is, the particle size of UiO-66-NH_2_, UiO@CeO_2_, and UiO@CeO_2_/IR@(bPEI/HA)-A6 was 150 ± 27.301nm, 162 ± 41.656 nm, and 165 ± 19.062 nm, respectively (Fig. 2E). The particle size was mainly determined by UiO-66-NH_2_ as the core, which was increased by coating with CeO_2_ and further increased by coating with (bPEI/HA)-A6. The particle size of UiO@CeO_2_/IR@(bPEI/HA)-A6 constructed in this study was less than 200 nm, which was beneficial to the material to penetrate and accumulate in the tumor site more effectively and play a therapeutic role [35]. The permeability and retention effect (EPR effect) allows materials with a particle size of less than 200 nm to enter the tumor tissue through the gap between the tumor vascular endothelium, and the lack of lymphatic reflux in the tumor tissue allows them to remain in the tumor tissue to achieve the purpose of targeted delivery of antitumor drugs [36].

DLS technology was used to measure the particle size of the DDS, namely, the particle sizes of UiO-66-NH_2_, UiO@CeO_2_, and UiO@CeO_2_/IR@(bPEI/HA)-A6 were approximately 248.0 ± 6.15 nm, 267.8 ± 3.08 nm, and 296.1 ± 4.07 nm, respectively (Fig. S2). Electrophoretic light scattering (ELS) was employed to measure the zeta potential. The average charges of UiO-66-NH_2_, UiO@CeO_2_, UiO@CeO_2_/IR, and UiO@CeO_2_/IR@(bPEI/HA)-A6 were 33.26 ± 2.23 mV, 26.16 ± 0.62 mV, −29.91 ± 0.45 mV, and −3.07 ± 0.78 mV, respectively (Fig. 2F). UiO-66-NH_2_ and UiO@CeO_2_ have positive charges, but IR-820 has a negative charge, and UiO@CeO_2_/IR shows a negative charge. The bPEI molecule contains an amino group and has a strong prophylactic property, so the surface of the modified material is generally positively charged. In contrast, the HA and A6 short peptides have negative charges. Finally, the negative charge of UiO@CeO_2_/IR@(bPEI/HA)-A6 is presented. Nanoparticles with negative surface charges are less toxic and more suitable for intravenous drug delivery, laying a foundation for their subsequent biomedical applications [37].

### Photothermal performance

IR-820 is a photosensitizer with high photothermal conversion efficiency, and its killing of tumor cells primarily depends on the large amount of thermal energy it produces under laser irradiation. Therefore, to investigate the photothermal conversion efficiency of UiO@CeO_2_/IR@(bPEI/HA)-A6, we used an 808-nm laser with an irradiation intensity of 1 W/cm^2^ to irradiate the nanomaterials. The results showed that UiO@CeO_2_/IR@(bPEI/HA)-A6 increased rapidly and reached a peak at 200 s. According to the heating and cooling curve, the photothermal conversion efficiency of the material was calculated to be 26.8%. The excellent photothermal properties of UiO@CeO_2_/IR@(bPEI/HA)-A6 were demonstrated (Fig. S3A).

Subsequently, the temperature changes of UiO@CeO_2_/IR@(bPEI/HA)-A6 at different concentrations in aqueous dispersions were monitored under 808-nm laser irradiation at 1 W/cm^2^ (Fig. S3B). The experiment demonstrated that the speed and degree of temperature increase increased continuously with increasing concentration. The photothermal properties of UiO@CeO_2_/IR@(bPEI/HA)-A6 are concentration dependent. When the concentration increases from 40 to 120 μg/ml, the temperature change within 5 min also increases, corresponding to the change, indicating that the photothermal efficiency of the nanocarrier is strong and also indicating the successful loading of IR-820. As a result, the photothermal properties of UiO@CeO_2_/IR@(bPEI/HA)-A6 nanoparticles were continuously enhanced with the increase of laser power density (Fig. S3C). In conclusion, UiO@CeO_2_/IR@(bPEI/HA)-A6 exhibits a promising photothermal effect, laying the groundwork for achieving a favorable photothermal therapeutic effect and effectively killing tumors.

### In vitro cell viability assay

**Fig. 3.**
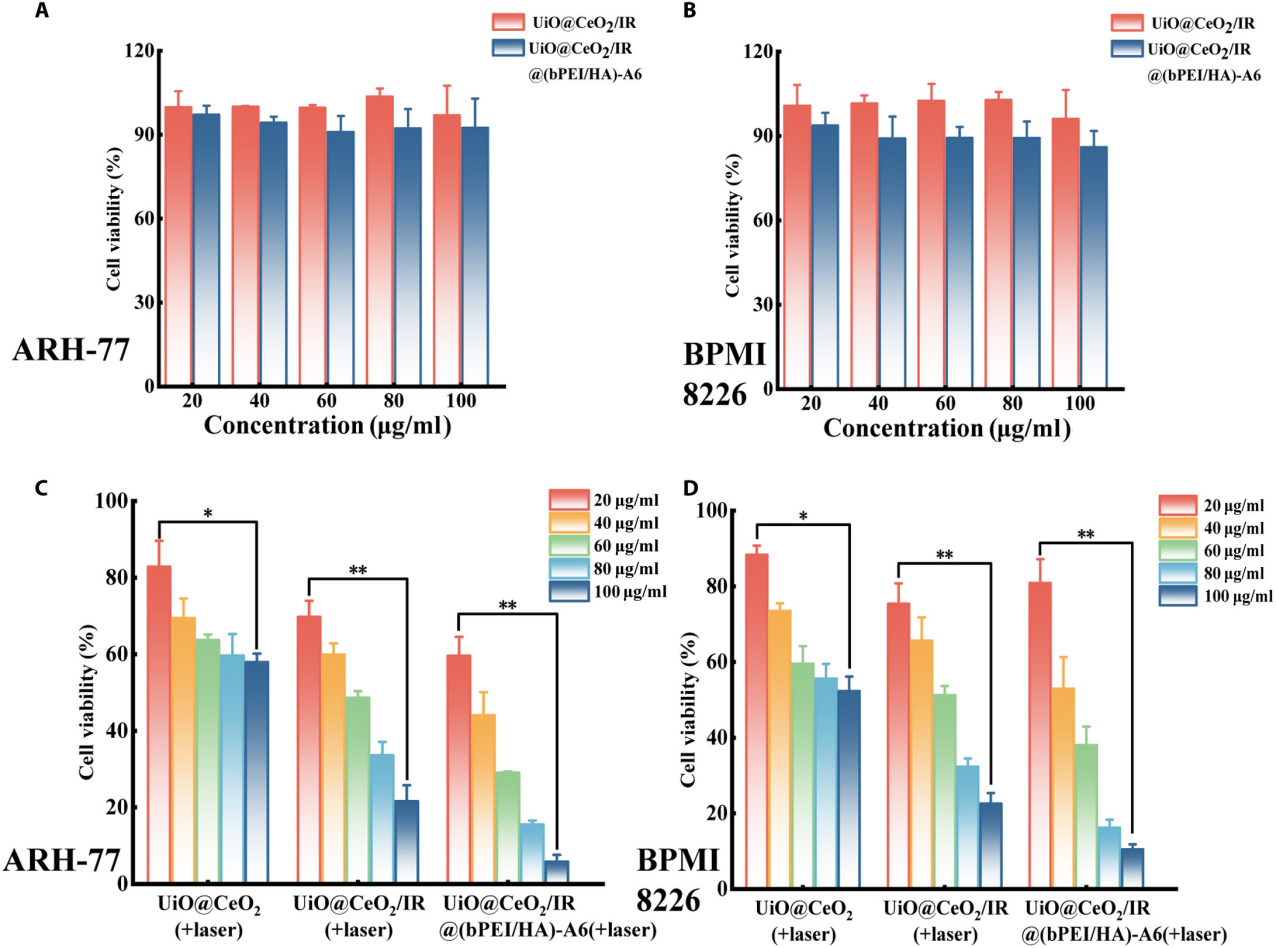
(A) The cell viability of ARH-77 cells following 24-h treatment with UiO@CeO_2_/IR and UiO@CeO_2_/IR@(bPEI/HA)-A6. (B) Cell viability of RPMI8226 cells following 24-h treatment with UiO@CeO_2_/IR and UiO@CeO_2_/IR@(bPEI/HA)-A6. (C) Cell viability of ARH-77 cells after being treated with different concentrations of UiO@CeO_2_, UiO@CeO_2_/IR, and UiO@CeO_2_/IR@(bPEI/HA)-A6, and then exposed to 808-nm laser (1 W/cm^2^). (D) Cell viability of RPMI8226 cells after being treated with different concentrations of UiO@CeO_2_, UiO@CeO_2_/IR, and UiO@CeO_2_/IR@(bPEI/HA)-A6, and then exposed to 808-nm laser (1 W/cm^2^). Data are presented as mean ± SD (*n* = 3) (*, **P < 0.05).

The dark toxicity and phototoxicity of UiO@CeO_2_/IR@(bPEI/HA)-A6 at the cellular level were characterized by CCK8 assay to evaluate its biosecurity and antitumor effect. For biosafety, ARH-77 and BPMI8226 cells were treated with UiO@CeO_2_/IR and UiO@CeO_2_/IR@(bPEI/HA)-A6 for 24 h, respectively, and cell viability was measured. Dark toxicity results under nonlaser conditions showed that cell viability was maintained above 80% in the studied concentration range (20 to 100 μg/ml) for UiO@CeO_2_/IR and UiO@CeO_2_/IR@(bPEI/HA)-A6, indicating good biosafety (Fig. 3A and B).

Appropriate 808-nm (1 W/cm^2^) laser irradiation can induce the nanotargeted DDS to exert photothermal killing ability. In terms of antitumor effect and targeting of drugs, ARH-77 and BPMI8226 cells were treated with UiO@CeO_2_, UiO@CeO_2_/IR, and UiO@CeO_2_/IR@ (bPEI/HA)-A6, respectively, and cell viability was measured after 808 nm (1 W/cm^2^) (Fig. 3C and D). Compared with the dark toxicity results, the phototoxicity results under 808-nm (1 W/cm^2^) laser irradiation showed that cell viability was slightly decreased after laser irradiation, even after the cells were treated with drugs without IR-820 loading. The cell viability was significantly reduced after laser irradiation, following treatment with medications loaded with IR-820. Moreover, the inhibition rate of myeloma cells increased with increasing drug concentration. At the same concentration, the cytotoxicity of UiO@CeO_2_/IR@(bPEI/HA)-A6 was significantly higher than that of UiO@CeO_2_/IR, all of which indicated that UiO@CeO_2_/IR@(bPEI/HA)-A6 had a good antitumor effect on myeloma cells. It can be further observed that at the same concentration, UiO@CeO_2_/IR@(bPEI/HA)-A6 exhibited a significantly better killing effect on ARH-77 cells with high CD44 expression compared to RPMI8226 cells with low CD44 expression. The results showed that UiO@CeO_2_/IR@(bPEI/HA)-A6 exhibited a good tumor-killing ability under laser irradiation and significantly inhibited the proliferation of ARH-77 cells, suggesting the promising application prospect and development potential of this nanotargeted DDS in antitumor research for multiple myeloma.

### Live/dead cells were stained to visualize cell survival

**Fig. 4.**
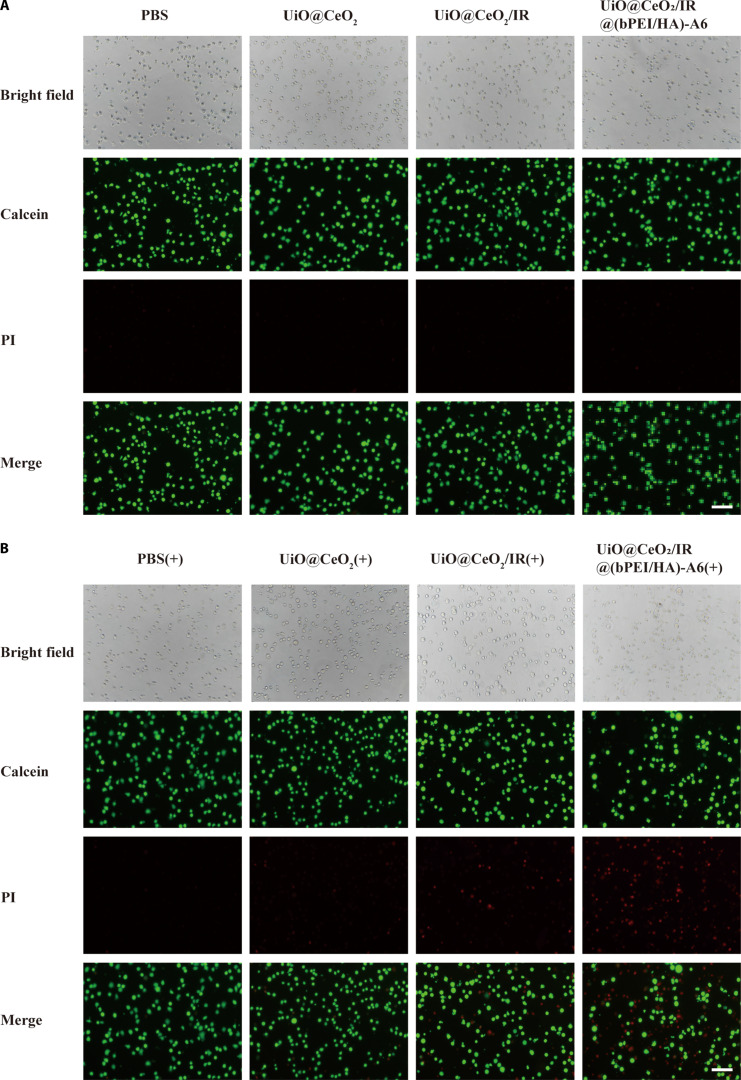
Stained images of live cells/dead cells after different treatments. (A) No 808-nm laser irradiation treatment. (B) An 808-nm laser irradiation treatment. Cell viability was observed through a fluorescence inverted microscope (scale bar, 100 μm).

Based on the good biocompatibility and photothermal properties of UiO@CeO_2_/IR@(bPEI/HA)-A6, we further investigated its inhibitory effect on tumor cells in vitro. Calcein-AM–PI live/dead cell staining is a fluorescent dye reagent that can stain live cells green and dead cells red, which can visually demonstrate the antitumor effect of the nanomedicine delivery system. By treating multiple myeloma ARH-77 cells with 2 large groups (nonlight group and light group) and 4 groups [PBS, UiO@CeO_2_, UiO@CeO_2_/IR, and UiO@CeO_2_/IR@(bPEI/HA)-A6], the growth of ARH-77 cells was observed under a fluorescence inverted microscope using calcein-AM–PI live/dead cell staining. The results showed that all groups that did not receive light exposure, as well as the group that received light exposure with PBS, exhibited the most green and least red fluorescence, indicating that these groups had a better cell state (Fig. 4A). It not only reflects biological safety but also allows cells to irradiate NIR light safely, with no cell-killing effect. For the cells treated with UiO@CeO_2_, UiO@CeO_2_/IR, and UiO@CeO_2_/IR@(bPEI/HA)-A6 in the light group, more red fluorescence appeared. The fluorescence increased gradually, indicating that the cell death rate also increased gradually under NIR light irradiation, and the tumor-killing effect of the nanotargeted DDS was gradually enhanced (Fig. 4B). In addition, UiO@CeO_2_/IR@(bPEI/HA)-A6 had the best killing effect on the tumor compared with other groups. The cell death rate increased sharply, indicating that A6 had a strong binding effect with CD44, and the targeting ability of the material was significantly enhanced.

### Cellular uptake

**Fig. 5.**
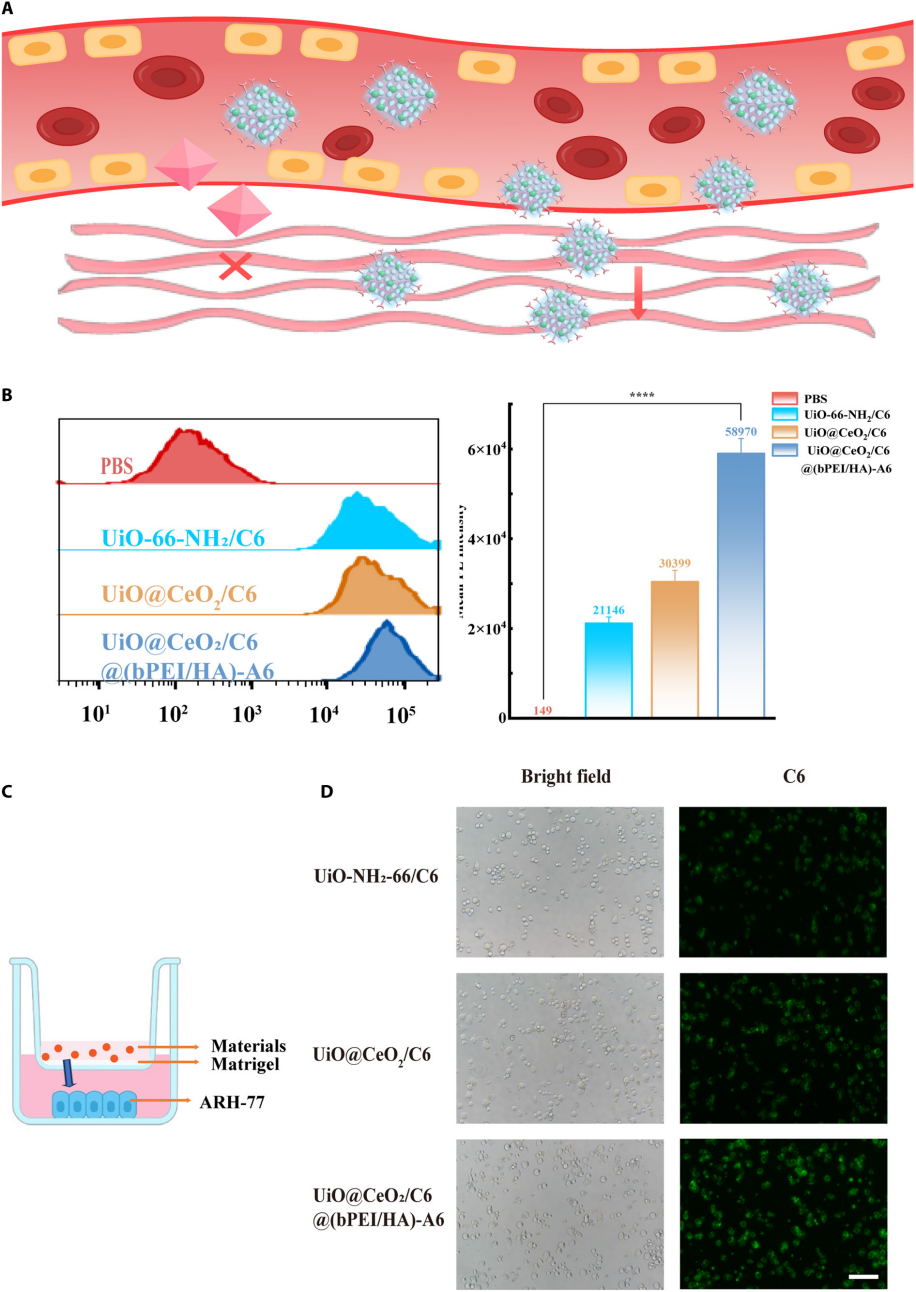
(A) Diagram showing the uptake of cells by different materials. (B) Treat ARH-77 cells with PBS, UiO-66-NH_2_/C6, UiO@CeO_2_/C6, and UiO@CeO_2_/C6@(bPEI/HA)-A6, incubate for 4 h, and then detect the uptake of the cells using a flow cytometer. (C) Transwell uptake experiment schematic diagram. (D) Treat ARH-77 cells with UiO-66-NH_2_/C6, UiO@CeO_2_/C6, and UiO@CeO_2_/C6@(bPEI/HA)-A6, incubate for 24 h, and then use a fluorescence inverted microscope to detect the uptake of cells (scale bar, 100 μm). Data are presented as mean ± SD (*n* = 3) (****P < 0.05).

To further investigate the better cellular uptake ability of the rough surface than the smooth surface of the material, the uptake of PBS, UiO-66-NH_2_/C6, UiO@CeO_2_/C6, and UiO@CeO_2_/C6@(bPEI/HA)-A6 by ARH-77 cells was investigated by flow cytometry. Compared with the PBS, UiO-66-NH_2_/C6, and UiO@CeO_2_/C6 groups, the fluorescence curve of the UiO@CeO_2_/C6@(bPEI/HA)-A6 group exhibited a significant rightward shift, indicating markedly enhanced cellular uptake of UiO@CeO_2_/C6@(bPEI/HA)-A6 by ARH-77 cells. Statistical analysis further revealed the specific fluorescence intensities of the 3 groups: The average fluorescence intensities in the PBS and UiO-66-NH_2_/C6 groups were 149 ± 6.08 and 221,146 ± 1,415.33, respectively, whereas that of the UiO@CeO_2_/C6 group was 58,970 ± 3,357.36 and the UiO@CeO_2_/C6@(bPEI/HA)-A6 group was 58,970 ± 3,357.36, which was substantially higher than those of the other 2 groups (Fig. 5A and B). These results further confirm that the cellular uptake of UiO@CeO_2_/C6@(bPEI/HA)-A6 is significantly greater than that of the PBS, UiO-66-NH_2_/C6, and UiO@CeO_2_/C6 groups. As the above electron microscopy has shown, the coating of CeO_2_ can make the surface of the nanomaterials rough, which further indicates that the rough surface can increase the uptake of nanodrugs, which can be more effective for the DDS to transport the drugs into the cells, provide a good drug concentration meter for tumor treatment, and further play a highly efficient tumor-killing effect.

### Transwell cell uptake

This study constructed a transwell coculture model to simulate the in vitro microenvironment of tumor cells and systematically evaluated the cellular uptake of 3 different nanomaterials [UiO-66-NH_2_/C6, UiO@CeO_2_/C6, and UiO@CeO_2_/C6@(bPEI/HA)-A6]. The experiments demonstrated that after 24 h of coculture, obvious cellular fluorescence signals were observed in all experimental groups, indicating that all 3 UiO-66-based nanomaterials could be effectively taken up by cells (Fig. 5C and D). Compared with UiO-66-NH_2_/C6, the fluorescence signals of UiO@CeO_2_/C6 and UiO@CeO_2_/C6@(bPEI/HA)-A6 were stronger. This rough surface increased the contact area and friction between the nanomaterials and the cell membrane, making them more easily captured by cell pseudopodia and thus passively enhancing the endocytosis of cells [38,39]. The UiO@CeO_2_/C6@(bPEI/HA)-A6 group showed the most vigorous cellular fluorescence intensity. The physical anchoring effect provided by the rough surface of CeO_2_, combined with the effective targeting property of bPEI/HA-A6, enables more efficient cellular uptake. UiO-66-NH_2_/C6 served as the basic control group, with a relatively smooth surface and relying solely on nonspecific endocytosis, thus having the lowest uptake efficiency. The experimental results further demonstrated that the surface properties of UiO@CeO_2_/C6@(bPEI/HA)-A6 were crucial in regulating its cellular uptake behavior. Not only were surface chemical modifications (such as the bPEI/HA-A6 coating) important, but their physical morphology (such as surface roughness) also played a significant positive role.

### Apoptosis analysis in vitro

**Fig. 6.**
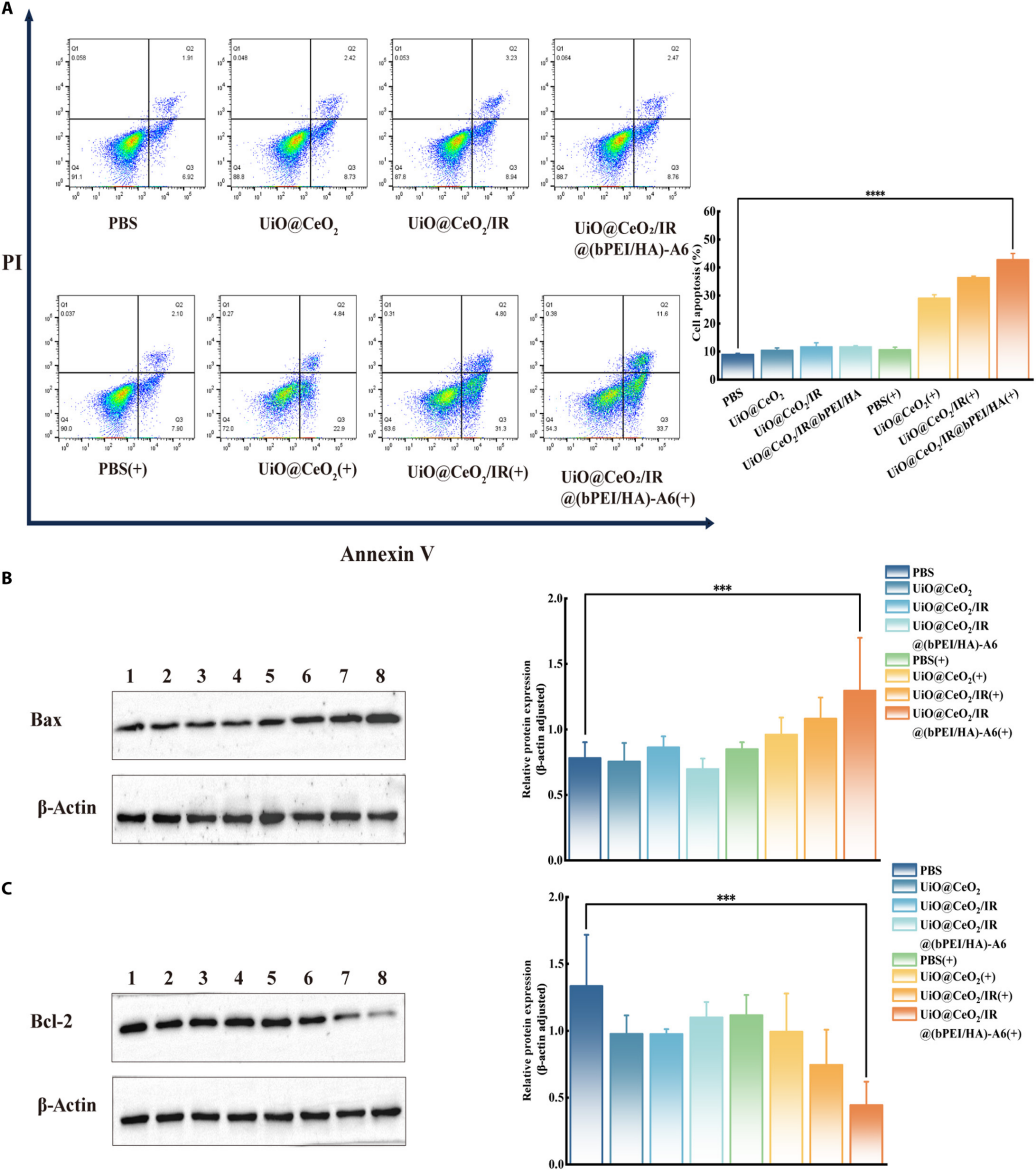
(A) Treat ARH-77 cells with PBS, UiO@CeO_2_, UiO@CeO_2_/IR, UiO@CeO_2_/IR@(bPEI/HA)-A6, PBS(+), UiO@CeO_2_(+), UiO@CeO_2_/IR(+), and UiO@CeO_2_/IR@(bPEI/HA)-A6(+) and measure cell apoptosis using flow cytometry. [Here, (+) indicates irradiation with an 808-nm laser (1 W/cm^2^).] (B and C) Protein expression of apoptosis-related genes in ARH-77 cells under different treatments. Note: 1, PBS; 2, UiO@CeO_2_; 3, UiO@CeO_2_/IR; 4, UiO@CeO_2_/IR@(bPEI/HA)-A6; 5, PBS(+); 6, UiO@CeO_2_(+); 7, UiO@CeO_2_/IR(+); 8, UiO@CeO_2_/IR@(bPEI/HA)-A6(+). Data are presented as mean ± SD (*n* = 3) (***, ****P < 0.05).

To further characterize the tumor-killing effect of UiO@CeO_2_/IR@(bPEI/HA)-A6, the apoptosis of ARH-77 cells was detected by Annexin V-FITC/PI apoptosis kit. ARH-77 cells were also divided into 2 large groups (the light group and the nonlight group), which included 4 groups [PBS, UiO@CeO_2_, UiO@CeO_2_/IR, and UiO@CeO_2_/IR@(bPEI/HA)-A6]. After 24 h of treatment, ARH-77 cells were detected by flow cytometry. The results showed that the cells in the nonlight group [PBS, UiO@CeO_2_, UiO@CeO_2_/IR, and UiO@CeO_2_/IR@(bPEI/HA)-A6] and the light group (PBS and UiO@CeO_2_) all had mild apoptosis. Among them, the apoptosis level in the PBS group of both the nonirradiation group and the irradiation group was lower. The apoptosis rates of PBS, UiO@CeO_2_, UiO@CeO_2_/IR, and UiO@CeO_2_/IR@(bPEI/HA)-A6 in the nonlight group were 8.98 ± 0.37%, 10.42 ± 0.83%, 11.67 ± 1.43%, and 11.66 ± 0.42%, respectively. The apoptosis rate in the PBS group was 10.65 ± 0.90%. The apoptosis rates of the light group UiO@CeO_2_, UiO@CeO_2_/IR, and UiO@CeO_2_/IR@(bPEI/HA)-A6 were 29.00 ± 1.26%, 36.39 ± 0.54%, and 42.77 ± 2.25%, respectively (Fig. 6A). Compared with the light group, the apoptosis level of the nonlight group was lower, and the apoptosis rate was less than 20%. The results showed that the UiO@CeO_2_/IR@(bPEI/HA)-A6 nanotargeted DDS itself had low toxicity and good biological safety. On the contrary, the apoptosis rate of UiO@CeO_2_, UiO@CeO_2_/IR, and UiO@CeO_2_/ IR@ (bPEI/HA)-A6 in the light group was higher, which was consistent with the trend verified by the above cell experiments. The apoptosis rate of the light group UiO@CeO_2_ was 29.00 ± 1.26%. This may be due to the pro-oxidation effect of CeO_2_ in the acidic environment of tumors, which induces apoptosis of tumor cells by generating ROS [40,41]. UiO@CeO_2_/IR@(bPEI/HA)-A6 showed the strongest cell-killing effect. Compared with other groups, the modification of the A6 short-peptide increased the targeting of the nano DDS and the more efficient killing of tumor cells.

### Analysis of the expression level of cell-related apoptosis proteins

To verify the specific molecules involved in apoptosis under drug treatment, the expression levels of apoptosis-related proteins Bcl-2 and Bax in ARH-77 cells following various treatments were evaluated using Western blot analysis. Two essential proteins involved in regulating apoptosis play a crucial role in controlling cell death. BAX belongs to the pro-apoptotic protein Bcl-2 family, while Bcl-2 is an anti-apoptotic protein [42]. The experimental results showed that, compared with the PBS group of the nonlight group, the differences between the 3 groups of UiO@CeO_2_, UiO@CeO_2_/IR, and UiO@CeO_2_/IR@(bPEI/HA)-A6 in the nonlight group and the PBS group of the light group were not particularly large. However, the differences in the 3 groups of UiO@CeO_2_, UiO@CeO_2_/IR, and UiO@CeO_2_/IR@(bPEI/HA)- A6 in the light group were particularly significant. The level of BAX gradually increased, and the level of Bcl-2 gradually decreased (Fig. 6B and C). Among them, UiO@CeO_2_/IR@(bPEI/HA)-A6 had the most significant effect, which can be further explained by UiO@CeO_2_/IR@(bPEI/HA)-A6 in the acidic environment of the tumor, where the coating of (bPEI/HA)-A6 was degraded, and the expression of Bax was increased, and Bcl-2 was decreased. Therefore, we can conclude that UiO@CeO_2_/IR@(bPEI/HA)-A6 can enhance the therapeutic effect of multiple myeloma in vitro and exhibits high biosafety, demonstrating effectiveness in killing cells, which has guiding significance for in vivo drug experiments.

### In vitro cell cycle analysis

**Fig. 7.**
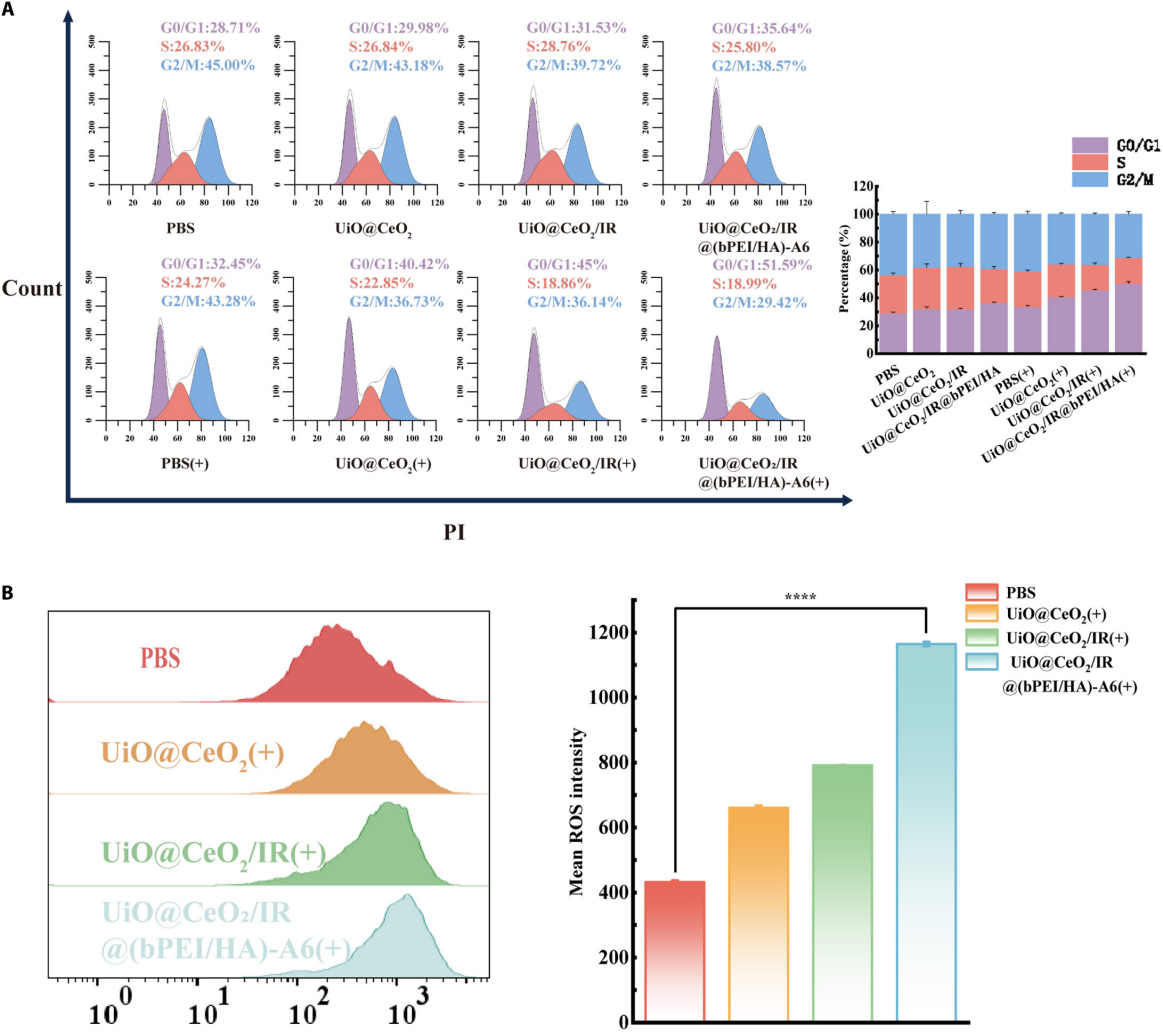
(A) Treat ARH-77 cells with PBS, UiO@CeO_2_, UiO@CeO_2_/IR, UiO@CeO_2_/IR@(bPEI/HA)-A6, PBS(+), UiO@CeO_2_(+), UiO@CeO_2_/IR(+), and UiO@CeO_2_/IR@(bPEI/HA)-A6(+) and then determine the cell cycle by flow cytometry. (B) Treat ARH-77 cells with PBS, UiO@CeO_2_(+), UiO@CeO_2_/IR(+), and UiO@CeO_2_/IR@(bPEI/HA)-A6(+) and detect the ROS content using a flow cytometer. Data are presented as mean ± SD (*n* = 3) (****P < 0.05).

The cell cycle is generally distinguished by its different phases, which are identified by changes in DNA content. ARH-77 cells were treated with the light group and nonlight group, which included 4 groups [PBS, UiO@CeO_2_, UiO@CeO_2_/IR, and UiO@CeO_2_/IR@(bPEI/HA)-A6]. The effect of different treatment methods on the cell cycle was analyzed by flow cytometry. The results are shown in the figure. The G0/G1 phase (the early stage of DNA synthesis) of the nonlight group [PBS, UiO@CeO_2_, UiO@CeO_2_/IR, and UiO@CeO_2_/IR@(bPEI/HA)-A6] and the light group (PBS) showed little change. However, the light group UiO@CeO_2_, UiO@CeO_2_/IR, and UiO@CeO_2_/IR@(bPEI/HA)-A6 showed G0/G1 phase arrest compared with the PBS group of the nonlight group, and the G0/G1 phase of UiO@CeO_2_ accounted for 40.64 ± 0.26%. The proportion of G0/G1 phase of UiO@CeO_2_/IR and UiO@CeO_2_/IR@(bPEI/HA)-A6 was 45.53 ± 0.64% and 50.09 ± 1.56%, respectively. Compared with the G0/G1 phase of the PBS group, the proportion of the G0/G1 phase in the PBS group was 29.01 ± 0.72%, indicating a significant cycle arrest. These results indicate that IR-820 can inhibit the synthesis of RNA and DNA after NIR light irradiation, and inhibit the proliferation of ARH-77 cells by inducing G0/G1 phase arrest. UiO@CeO_2_ did not load with IR-820 but still blocked the G0/G1 phase of ARH-77 cells (Fig. 7A). The primary reason was that the rough surface of CeO_2_ enhanced cell uptake, and CeO_2_ also promoted the apoptosis of tumor cells. UiO@CeO_2_/IR@(bPEI/HA)-A6 was more specific than the light group UiO@CeO_2_ and UiO@CeO_2_/IR. This is because the polyelectrolyte encapsulation of IR-820 prevents the leakage of the drug and the strong binding of A6 short peptide to the CD44 overexpressed on the surface of ARH-77 leads to the improvement of drug transport efficiency and targeting so that the drug can be accumulated in the tumor tissue.

### ROS detection

In this experiment, DHE red fluorescent dye was used to detect the content of superoxide anion ROS in cells. DHE can freely enter the cell through the living cell membrane and be oxidized by intracellular ROS to form ethidium oxide, which can be incorporated into chromosomal DNA to produce red fluorescence, which can be further detected by flow cytometry. The figure shows the ROS generation after PBS, UiO@CeO_2_, UiO@CeO_2_/IR, and UiO@CeO_2_/IR@(bPEI/HA)-A6 treatment for 24 h and NIR light irradiation. Compared with PBS group ROS intensity 432 ± 5.57, the ROS intensity produced by UiO@CeO_2_, UiO@CeO_2_/IR, and UiO@CeO_2_/IR@(bPEI/HA)-A6 was 664.00 ± 7.00, 791.33 ± 1.52, and 1,164.33 ± 7.02, respectively. Results showed that the ROS level increased gradually (Fig. 7B). The results showed that UiO@CeO_2_ could also produce ROS under NIR light irradiation, which was attributed to CeO_2_ acting as a pro-oxidant in the weak acid microenvironment of the tumor, thereby producing ROS (including superoxide anions) and promoting oxidative damage to tumor cells. In addition, UiO@CeO_2_/IR@(bPEI/HA)-A6 produced more ROS after laser irradiation, which fully indicated that UiO@CeO_2_/IR@(bPEI/HA)-A6 exposed IR-820 under acidic tumor conditions, and the rough surface increased the uptake of materials by cells. The photothermal properties were stimulated to produce more ROS [43]. When combined with DHE, it showed fluorescence. Furthermore, we can conclude that UiO@CeO_2_/IR@(bPEI/HA)-A6 exhibits good ROS generation ability, photothermal performance, and cellular uptake ability and can be actively utilized for the treatment of multiple myeloma cells.

### In vivo biodistribution of nanotargeted DDS

**Fig. 8.**
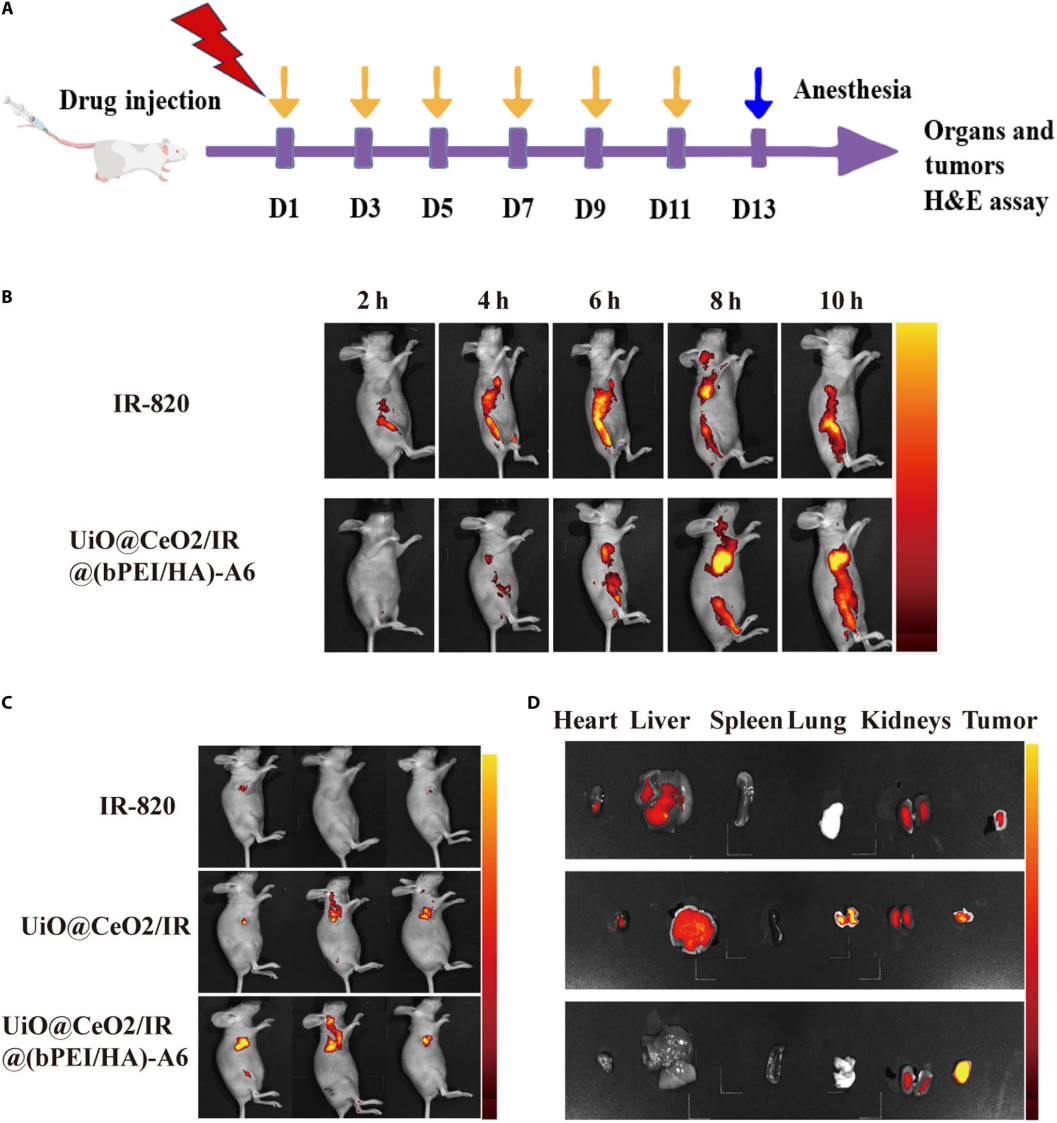
(A) Flowchart of the treatment process for mice. (B) Real-time distribution of IR-820 and UiO@CeO_2_/IR@(bPEI/HA)-A6 in tumor sites of tumor-bearing mice. (C) Fluorescence images of BALB/c mice 24 h after intravenous injection of IR-820, UiO@CeO_2_/IR, and UiO@CeO_2_/IR@(bPEI/HA)-A6. (D) Fluorescent imaging of major organs and tumors in mice after tail vein injection for 24 h.

To evaluate the accumulation ability of the nanotargeted DDS in the tumor site and determine the appropriate time point for laser irradiation of the tumor site after intravenous injection, a small animal in vivo imaging system was used to detect the fluorescence distribution of IR-820 itself in mice after administration of different groups [IR-820 and UiO@CeO_2_/IR@(bPEI/HA)-A6]. The fluorescence signals at various time points (2, 4, 6, 8, and 10 h) after intravenous injection of different groups of drugs into the mice were detected (Fig. 8A and B). According to the fluorescence signal of the tumor site, the fluorescence intensity of the tumor site in the UiO@CeO_2_/IR@(bPEI/HA)-A6 group was significantly higher than that in the IR-820 group at several time points, which indicated that UiO@CeO_2_/IR@(bPEI/HA)-A6 had good tumor accumulation and penetration ability. This may be related to the targeting effect of the A6 short peptide and the increased uptake of CeO_2_ by tumor cells. In addition, it was observed that the fluorescence signal in the liver at each time point after administration was significantly enhanced compared with the free IR-820 group, which may be due to the liver as a metabolic organ. UiO@CeO_2_/IR@(bPEI/HA)-A6 entered the body circulation and was excreted through the liver metabolism. Because the fluorescence intensity of the tumor in the UiO@CeO_2_/IR@(bPEI/HA)-A6 group was the highest at 8 h after drug injection, to achieve the maximum degree of drug accumulation in the tumor site to achieve the best antitumor effect in the following experiments, 8 h after drug injection was used as the best laser irradiation time for the experiment.

### UiO@CeO_2_/IR@(bPEI/HA)-A6 targeting in vivo

To explore the targeting ability of UiO@CeO_2_/IR@(bPEI/HA)-A6 in vivo, the biodistribution of IR-820 in mice was examined 24 h after intraperitoneal injection. According to the fluorescence signal of the tumor site in mice, the fluorescence intensity of the tumor site in the UiO@CeO_2_/IR@(bPEI/HA)-A6 group was significantly higher than that in the IR-820 and UiO@CeO_2_/IR groups, indicating that the A6 short peptide and HA had better targeting ability to CD44. This further indicated that UiO@CeO_2_/IR@(bPEI/HA)-A6 had a good targeting ability to the tumor in vivo (Fig. 8C).

After that, mice with tumors were anesthetized and killed, and tumors and vital organs (heart, liver, spleen, lung, and kidney) were harvested for overfluorescence intensity detection. Compared with the IR-820 group, the fluorescence intensity of the tumor in UiO@CeO_2_/IR@(bPEI/HA)-A6 group still maintained a high level 24 h after injection, followed by UiO@CeO_2_/IR, further demonstrating that UiO@CeO_2_/IR@(bPEI/HA)-A6 has good tumor targeting and tumor accumulation ability (Fig. 8D). This study provides a solid foundation for achieving an excellent therapeutic effect against tumors in vivo. Comparing the fluorescence signals of other significant organs in the UiO@CeO_2_/IR and IR-820 groups, it was found that the fluorescence intensity of each organ in the UiO@CeO_2_/IR group was higher than that in the IR-820 group, which indicated that the hydrophilic drug IR-820 was easily metabolized and removed in the body. At the same time, UiO@CeO_2_/IR could achieve longer time accumulation in the tumor site. The fluorescence intensity of liver and kidney in the UiO@CeO_2_/IR group was stronger than that in other groups, which may be related to the metabolism of materials. The fluorescence signal in the liver may be due to the immune evasion ability of the UiO@CeO_2_/IR group. The construction of nanomedicines enhances the stability of drug metabolism in vivo compared to conventional drugs. Still, excessive drug accumulation in nontumor sites may lead to toxic side effects in normal tissues. Except for the kidney, the principal organs of the UiO@CeO_2_/IR@(bPEI/HA)-A6 group exhibited a lower fluorescence signal, which was attributed to the targeting ability of UiO@CeO_2_/IR@(bPEI/HA)-A6. The above results indicate that UiO@CeO_2_/IR@(bPEI/HA)-A6 exhibits good targeting and drug accumulation abilities in tumor sites, laying a solid foundation for subsequent tumor treatment effects.

### UiO@CeO_2_/IR@(bPEI/HA)-A6 antitumor effect and biosafety in vivo

**Fig. 9.**
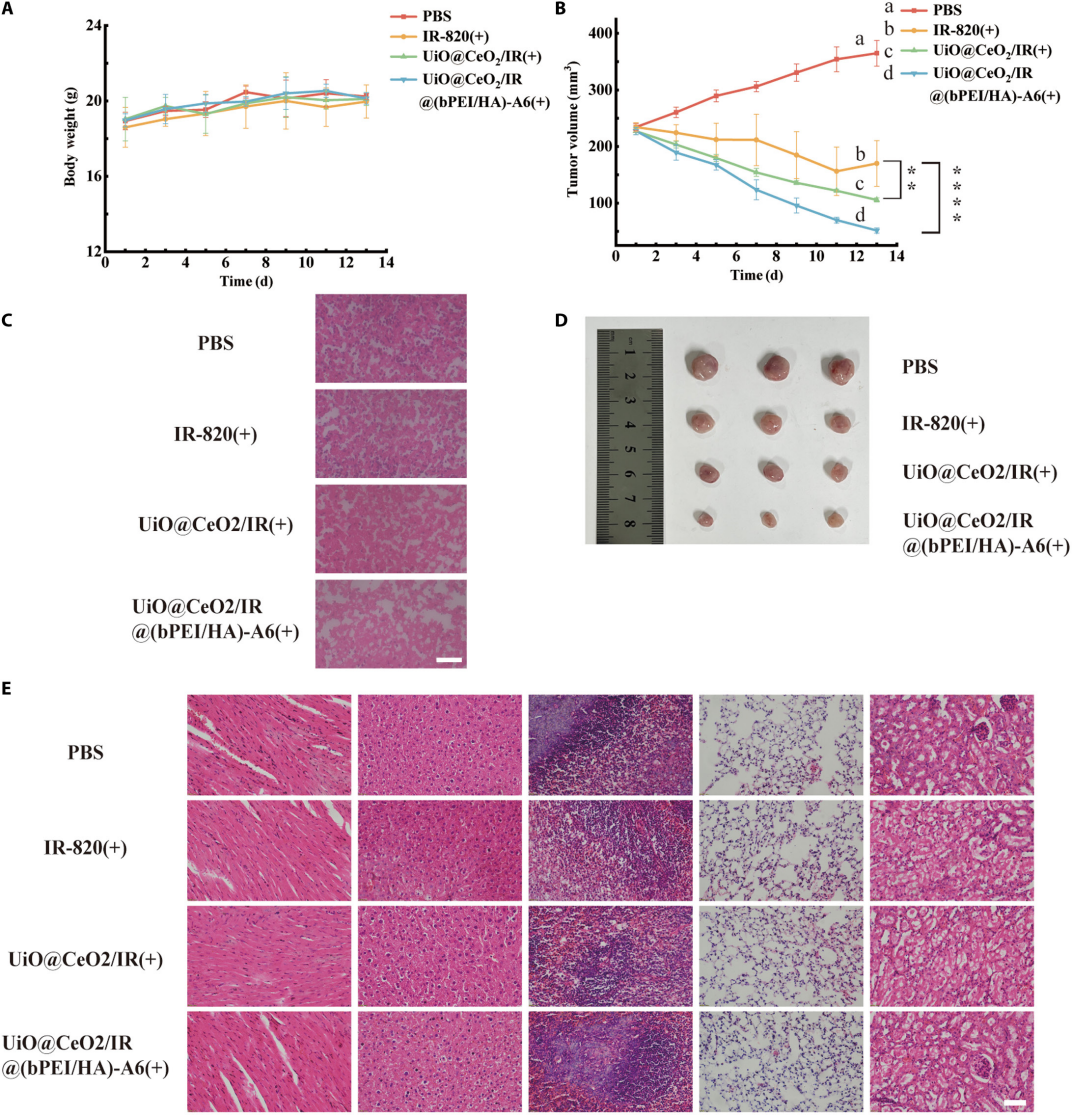
(A) Changes in the body weight of mice in different treatment groups. (B) Changes in tumor volume of mice in different treatment groups. (C) H&E staining analysis of tumor tissues in nude mice treated under various conditions (Olympus microscope; 40× magnification; scale bar, 50 μm). (D) Tumor mass images captured on the 14th day. (E) H&E analysis of primary organ tissues of BALB/c nude mice treated under different conditions. Data are presented as mean ± SD (*n* = 3) (**, **** *P* < 0.05).

To study the antitumor effect of UiO@CeO_2_/IR@(bPEI/HA)-A6 in vivo, the tumor inhibitory effect of UiO@CeO_2_/IR@(bPEI /HA)-A6 in mice was studied. Compared with PBS, IR-820, and UiO@CeO_2_/IR, UiO@CeO_2_/IR@(bPEI/HA)-A6 has a better accumulation effect in tumor sites and plays a good tumor growth inhibition effect. During the entire treatment period, changes in body weight and tumor growth were observed. During the observation, the body weight of the mice did not change significantly (Fig. 9A), while the tumor volume of IR-820, UiO@CeO_2_/IR, and UiO@CeO_2_/IR@(bPEI/HA)-A6 showed a downward trend, and UiO@CeO_2_/IR@(bPEI/HA)-A6 showed the most significant decrease (Fig. 9B). These results indicated that the above materials had good biological safety and excellent antitumor ability. Three mice in each group were sacrificed under anesthesia, and the tumor masses were collected and photographed. The tumor volume of UiO@CeO_2_/IR@(bPEI/HA)-A6 was smaller than that of the other 3 groups (Fig. 9D).

To further demonstrate the antitumor effect and biosafety of UiO@CeO_2_/IR@(bPEI/HA)-A6 in vivo, major organs (heart, liver, spleen, and lung, kidney) and tumor tissues were evaluated by hematoxylin and eosin (H&E) staining. The results showed that the tumor tissue of the UiO@CeO_2_/IR@(bPEI/HA)-A6 group exhibited extensive necrosis and apoptosis compared to the other 3 groups, demonstrating its practical tumor-killing ability (Fig. 9C). In the biosafety test, no apparent toxic damage or inflammation was found in the tissue sections of the heart, liver, spleen, lung, and kidney of mice. The results showed that UiO@CeO_2_/IR@(bPEI/HA)-A6 nanoparticles did not cause harm to mice and exhibited high biological safety, providing a safety guarantee for their application in tumor therapy (Fig. 9E). Taken together, the enhanced antitumor effects of UiO@CeO_2_/IR and UiO@CeO_2_/IR@(bPEI/HA)-A6 compared with free IR-820 indicated that the constructed DDS was more stable. UiO@CeO_2_/IR@(bPEI/HA)-A6 had a good targeting ability compared with the other 3 groups and significantly inhibited the tumor growth without abnormal organ pathological changes, indicating that the drug was safe at the current dose.

## Conclusion

In this study, we modified the rough surface of CeO₂ with UiO-66-NH₂ and then loaded the drug IR-820. Finally, we constructed the UiO@CeO_2_/IR@(bPEI/HA)-A6 drug delivery system by surface coating with bPEI and HA-A6. This strategy enhances the targeting efficacy of the drug, promotes increased accumulation at the tumor site, and facilitates enhanced cellular uptake of the nanocarrier. In vitro cell viability assays and live/dead cell staining demonstrated the cytotoxicity of DDS under 808-nm laser irradiation, proving that the combination of CeO_2_ and HA-A6 exhibits excellent targeting ability and cell uptake capability. The results of cell apoptosis and related proteins indicated that DDS exhibited a good antitumor effect in the cell apoptosis mechanism. In vivo studies showed that DDS has excellent targeting ability and an outstanding tumor treatment effect. It was shown that 24 h after intravenous injection, the accumulation of UiO@CeO_2_/IR@(bPEI/HA)-A6 in the tumor site was significantly higher than in other organs, indicating its good targeting effect in the tumor site. H&E staining verified the good tumor-killing ability and biological safety of DDS.

The enhancement of drug targeting is achieved by the specific binding of HA and A6 short peptides to CD44, and the enhancement of cellular uptake is achieved by the synthesis of the rough surface. On the one hand, UiO@CeO_2_/IR@(bPEI/HA)-A6 exhibits good biosafety both in vivo and in vitro, making it a good nanotargeted DDS. On the other hand, UiO@CeO_2_/IR@(bPEI/HA)-A6 can degrade the pH-responsive polyelectrolyte shell in the acidic TME, thereby exposing the drug and inducing a photothermal effect, which promotes the PDT of CeO_2_. It shows good nanotargeted DDS and antitumor ability in vivo and in vitro. UiO@CeO_2_/IR@(bPEI/HA)-A6 exhibits a relatively simple preparation process. It demonstrates good tumor-targeting ability, as well as low toxic side effects, in both in vivo and in vitro studies, resulting in a promising therapeutic impact on multiple myeloma. This study presents a potential approach for treating CD44 overexpression in multiple myeloma using near-infrared laser therapy, offering broader insights and additional therapeutic strategies for new multiple myeloma treatments.

## Data Availability

The authors do not have permission to share data.

## Supplementary Materials

Supplementary 1 Figs. S1 to S3 Graphical Abstract
