# Supplementary material for: Construction of Rough Surfaces Based on Zirconium Metal–Organic Frameworks to Enhance Photothermal and Photodynamic Therapy for Multiple Myeloma
Source: Biomater Res. 2026 Feb 20;30:0330. doi: 10.34133/bmr.0330 (PMC12921127; doi:10.34133/bmr.0330)
Supplement: Supplementary 1 — Figs. S1 to S3 Graphical Abstract [file bmr.0330.f1.docx]

Figure S1 FT-IR spectra of the UiO@CeO_2_, and UiO@CeO_2_/IR.





Figure S2 The peak values of the light intensity distribution of the particle size calculated by dynamic light scattering curves for UiO-66-NH_2_, UiO@CeO_2_, and UiO@CeO_2_/IR@(bPEI/HA)-A6.


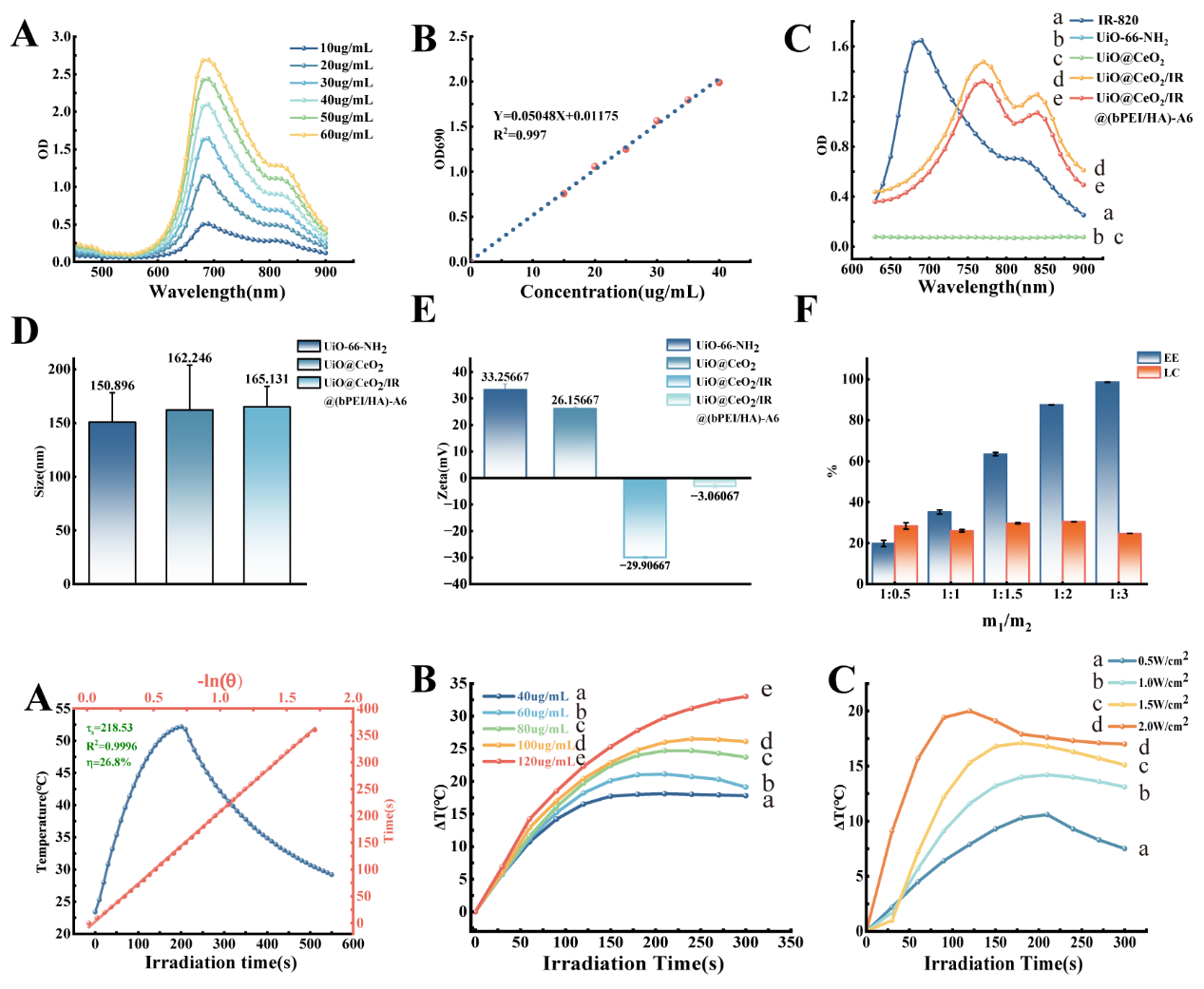
Figure S3 (A) Temperature change in the solution of UiO@CeO_2_/IR@(bPEI/HA)-A6 (100 μg/mL) by turning the NIR laser on and off (808 nm, 1.0 W/cm^2^), and a plot of -ln(θ) versus time obtained from the cooling period for 550 s. (B) Concentration-dependent photothermal effect of UiO@CeO_2_/IR@(bPEI/HA)-A6 under 808 nm irradiation at 1.0 W/cm^2^. (C) Temperature elevation curves upon different 808 nm laser power densities.
